# *NvPrdm14d*-expressing neural progenitor cells contribute to non-ectodermal neurogenesis in *Nematostella vectensis*

**DOI:** 10.1038/s41467-023-39789-4

**Published:** 2023-08-10

**Authors:** Quentin I. B. Lemaître, Natascha Bartsch, Ian U. Kouzel, Henriette Busengdal, Gemma Sian Richards, Patrick R. H. Steinmetz, Fabian Rentzsch

**Affiliations:** 1 Michael Sars Centre, University of Bergen, Thormøhlensgate 55, 5006 Bergen, Norway; 2 Department for Biological Sciences, University of Bergen, Thormøhlensgate 55, 5006 Bergen, Norway

**Keywords:** Neurogenesis, Evolutionary developmental biology, Differentiation

## Abstract

Neurogenesis has been studied extensively in the ectoderm, from which most animals generate the majority of their neurons. Neurogenesis from non-ectodermal tissue is, in contrast, poorly understood. Here we use the cnidarian *Nematostella vectensis* as a model to provide new insights into the molecular regulation of non-ectodermal neurogenesis. We show that the transcription factor *NvPrdm14d* is expressed in a subpopulation of *NvSoxB(2)-*expressing endodermal progenitor cells and their *NvPOU4*-expressing progeny. Using a new transgenic reporter line, we show that *NvPrdm14d*-expressing cells give rise to neurons in the body wall and in close vicinity of the longitudinal retractor muscles. RNA-sequencing of *NvPrdm14d*::GFP-expressing cells and gene knockdown experiments provide candidate genes for the development and function of these neurons. Together, the identification of a population of endoderm-specific neural progenitor cells and of previously undescribed putative motoneurons in *Nematostella* provide new insights into the regulation of non-ectodermal neurogenesis.

## Abstract (web summary)

Endodermal neurogenesis is an uncommon trait among animals. Here they identify a population of endoderm-specific neural progenitor cells in the cnidarian Nematostella vectensis that is characterized by the expression of the transcription factor prdm14d.

## Introduction

**Fig. 1:**
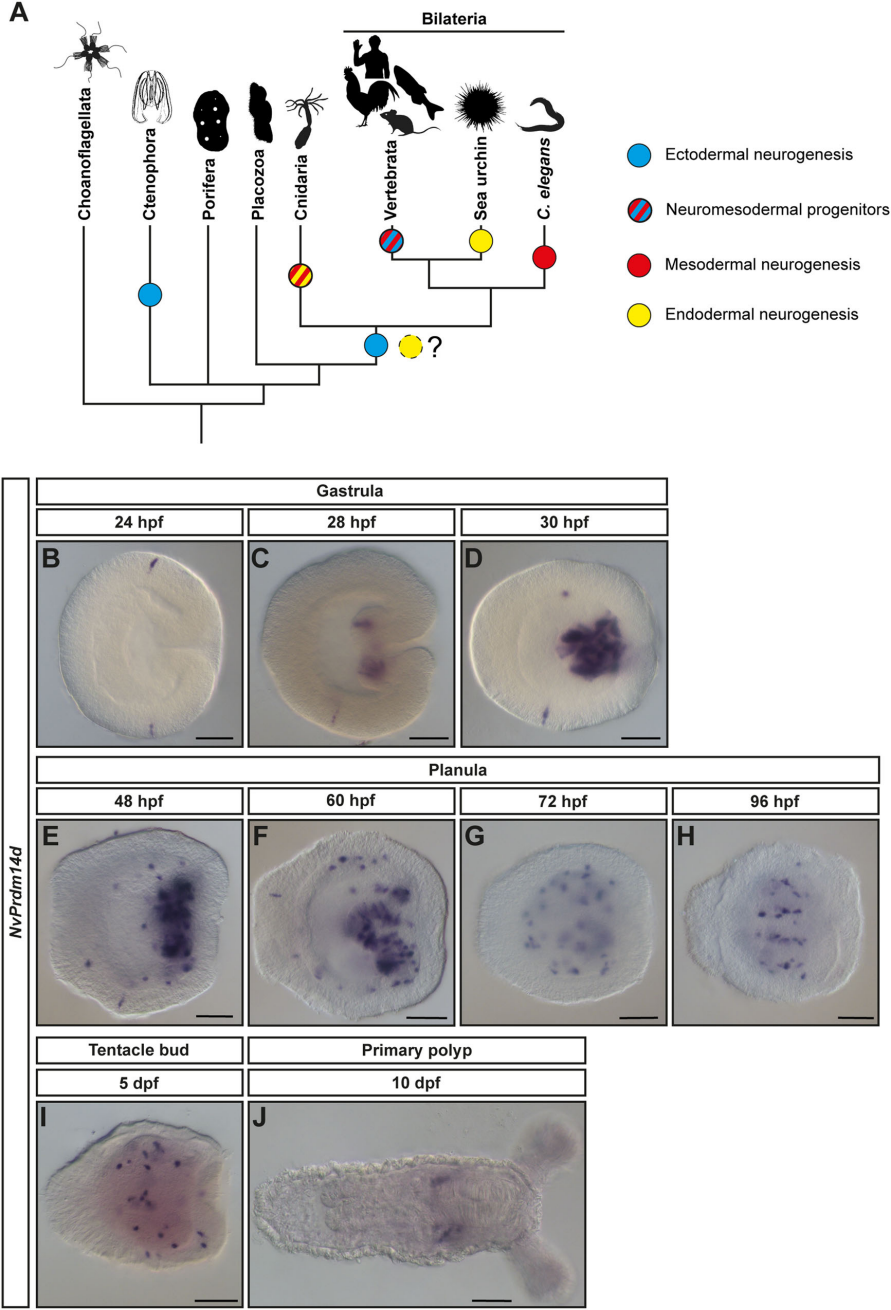
*NvPrdm14d* is mainly expressed in scattered endodermal cells. **A** Simplified phylogeny showing the distribution of ectodermal and non-ectodermal neurogenesis across metazoans. Presence of a nervous system is indicated by a blue dot, independently from a single or multiple origin(s) of the nervous system. Non-ectodermal neurogenesis is indicated with dots of various colors illustrating the germ layer producing non-ectodermal neurons and it is restricted to examples for which molecular data are available. Except for hydrozoan cnidarians, all cases of non-ectodermal neurogenesis are occurring in addition to ectodermal neurogenesis. Red and yellow colors for cnidarians indicate that the homology of the internal germ layer is debated. When applicable, cartoons show representative species in which non-ectodermal neurogenesis has been studied. The phylogeny is rooted with choanoflagellates as outgroup and is based on ref. ^100^. Animal silhouettes are from https://phylopic.org/. **B**–**J** Expression pattern of *NvPrdm14d* by colorimetric in situ hybridization. **B** In the early gastrula, *NvPrdm14d* is expressed in very few single ectodermal cells. **C**, **D** In mid-gastrula, *NvPrdm14d* starts to be expressed in the pharynx. Strong expression in the pharynx persists until mid-planula (**F**). In early planula (**E**), *NvPrdm14d* starts to be expressed in scattered endodermal cells. **G**–**I** From mid-planula, the expression remains in scattered cells within the pharynx and the endoderm. **J** In the primary polyp, *NvPrdm14d* is expressed in lateral domains within the endoderm. In all pictures, the oral pole is oriented to the right. Scale bars: 50 µm.

Adult animals contain neural cells located in tissues derived from all embryonic germ layers whereas neurogenesis, the generation of these neural cells, occurs in most animals almost exclusively from neural progenitor cells of ectodermal origin. While non-ectodermal neurogenesis occurs in several taxa (Fig. 1A), it typically contributes only a small fraction of neurons. Even the non-ectodermal (*i.e*. mesodermal and endodermal) aspects of the nervous system mainly consist of neurons generated by neural progenitors or precursors of ectodermal origin that migrated into these germ layers^1–3^. The molecular regulation of non-ectodermal neurogenesis has been studied in only a few cases and it remains therefore unclear to what extent ectodermal and non-ectodermal neurogenesis differ within a given species, and whether conserved regulators of non-ectodermal neurogenesis exist^4–7^.

In vertebrates, neuromesodermal progenitors (NMps) are bipotent cells located in the tailbud and they are involved in axial elongation^4^. These cells are presumably derived from mesodermal tissues and have the potential to produce both the posterior spinal cord and paraxial mesoderm^8^. The bipotency of these NMps is maintained by the co-expression of the neural marker gene *Sox2* and the early mesodermal marker gene *T/Brachyury*^9–11^. NMps are specified to either a neural or paraxial mesoderm fate depending on the extrinsic cues they receive when leaving their niche. Anteriorly, cells are exposed to high levels of retinoic acid and initiate neural differentiation, while posteriorly, they are exposed to high levels of Wnt3a and initiate mesodermal differentiation^9,12–18^. These NMps contribute to posterior neural structures and to the posterior somites^19,20^.

In the nematode *Caenorhabditis* *elegans*, 6 out of the 20 pharyngeal neurons are derived from the mesodermal lineage MS, including the I4 neurons^21^. It has been shown that the I4 neurons are specified in part through the action of the proneural protein HLH-3 and the HLH-2/Mediator complex repressing the CDK-7/Cyclin-H complex^5^. However, the specification of the other mesoderm-derived neurons remains to be investigated.

In the sea urchin, the endodermal origin of foregut neurons has been demonstrated by using photoconvertible fluorescent proteins^7^. In the endoderm, the expression of *SoxB1* maintains the neurogenic potential and induces the expression of *Six3*. Then, *Six3* activates the *SoxC*-mediated and *Nkx3-2*-mediated neurogenic cascades. While the *SoxC* cascade is used in both ectodermal and endodermal neurogenic pathways, the *Nkx3-2* cascade seems to be specific to the endodermal neurogenic pathway. It is also apparent that Nodal, BMP, Notch, FGF and Wnt signaling are involved in sea urchin endodermal neurogenesis, but further investigations are required to understand their respective interactions within the endodermal neurogenic pathway^7,22–24^.

Cnidarians, the sister group to bilaterians, develop from only two germ layers, here called ectoderm and endoderm. The cnidarian endoderm has been suggested to be homologous to either both endoderm and mesoderm, or only to the mesoderm of bilaterians^25–27^ and it gives rise to tissue that is lining the gastric cavity (the gastrodermis). The cnidarian ectoderm gives rise to the epidermis and in anthozoan cnidarians also to parts of the gastrodermis. The gastrodermis of anthozoan cnidarians further includes the pharynx, which consists of endoderm-derived and ectoderm-derived parts. The nervous system of cnidarian polyps lacks brain-like centralization and consists of sensory/sensory-motor neurons, ganglion neurons (morphologically similar to interneurons) and cnidocytes, the phylum-specific stinging cells^28–31^. Non-ectodermal neurogenesis is a common feature in cnidarians. Hydrozoan cnidarians, such as *Hydra* and *Hydractinia*, generate the entirety of their nervous system from an endoderm-derived cell type: the interstitial cells^32–38^. In anthozoan cnidarians, notably the sea anemone *Nematostella vectensis*, the nervous system is found in both the epidermis and the gastrodermis^31,39,40^. We will here refer to neurogenesis in the outer layer at gastrula and planula stages as ectodermal, and to neurogenesis in the inner layer of the body wall at planula stage as endodermal. For the pharynx we use the terms ectodermal and endodermal pharynx at gastrula and planula stages, and we consider it as gastrodermal tissue from primary polyp stage on. We will refer to neurons from primary polyp stage on as epidermal or gastrodermal. In *Nematostella*, the endodermal origin of endodermal/gastrodermal neurons in the body wall of the planula larva and primary polyp has been demonstrated by transplantation experiments^39^. Progenitors that give rise to neurons can first be detected at blastula stage and at the end of gastrulation also in the inner tissue layer^40^. Perturbation experiments showed that Notch signaling and the transcription factors *NvAth-like* and *NvSoxB(2)* act at an early stage of both ectodermal and endodermal neurogenesis, with Notch signaling acting as a negative and *NvAth-like* and *NvSoxB(2)* as positive regulators, respectively^6,40,41^. Downstream of these factors, *NvAshA*, *NvDmrtB* and *NvPOU4* were shown to be expressed and function in large populations of ectodermal/epidermal and endodermal/gastrodermal neural cells^42–44^. However, the molecular mechanisms underlying more specifically endodermal neurogenesis in *Nematostella* have not been investigated so far. In the present study, we show that *NvPrdm14d* is involved in the generation of a subpopulation of endodermal/gastrodermal neurons.

Prdm14 belongs to the PR domain (PRDI-BF1 and RIZ1 homology domain) containing family of transcription factors^45–47^. Prdm proteins are composed of a PR domain related to the catalytic SET domain (Suppressor of variegation 3–9, Enhancer of zeste and Trithorax) characterizing a large group of histone lysine methyltransferases (HMT), followed by a variable number of zinc fingers at the C-terminus^46,48–52^. While the PR domain of Prdm14 does not show intrinsic HMT activity, it recruits co-factors, whereas the zinc fingers directly bind to regulatory elements of target genes^53–56^.

In vertebrates, Prdm14 is a major factor involved in the maintenance of embryonic stem cells. It recruits TET (Ten-Eleven Translocation protein) to the promoter of pluripotency genes (*e.g. Pou5f1/Oct4*, *Nanog*, *Sox2*, *Klf5*) for promoting their expression, while in parallel, it recruits PRC2 (Polycomb Repressive Complex 2) for repressing differentiation genes^53,55–60^. Furthermore, Prdm14 can function in the reprogramming of differentiated cells to induced pluripotent stem cells (iPS) in vitro^53,61–64^.

Similarly, Prdm14 is required for the specification of primordial germ cells (PGCs) in vertebrates as it allows the reacquisition of pluripotency. It acts in synergy with Prdm1 and TFAP2C (encoding AP2γ) to promote pluripotency and germline-specific gene expression, while repressing somatic genes^63,65–67^. The absence of Prdm14 leads to a deficiency of PGCs and therefore results in sterility^66–70^.

In the zebrafish, Prdm14 is involved in axon outgrowth of primary motoneurons through the activation of *Islet2*^54^. A role for *Prdm14* and *Islet2* in the development of motoneurons has also been described in amphioxus, a non-vertebrate chordate^71^. These observations led to the suggestion that the ancestral function of *Prdm14* is in neural development and that it has been co-opted for the regulation of pluripotent cells during vertebrate evolution^71^.

In the present report, we identify *NvPrdm14d* as a factor involved in endodermal neurogenesis in *Nematostella*. By combining colorimetric and fluorescent in situ hybridization with EdU labeling, we show that *NvPrdm14d* is expressed in a subpopulation of endodermal neural progenitor cells (NPCs), as well as in the progeny of these cells. We generated a *NvPrdm14d::GFP* transgenic reporter line, which highlights a population of putative motoneurons in the close vicinity of retractor muscles. We then sorted *NvPrdm14d*::GFP-expressing cells and generated their transcriptome to identify additional potential candidate genes involved in the generation of such neurons and tested the requirement of *NvPrdm14d* for their expression by shRNA injection.

## Results

### *NvPrdm14d* is a candidate gene involved in endodermal neurogenesis

The genome of *N. vectensis* contains four well conserved *Prdm14* paralogs [*NvPrdm14a-d*^72^,]. Inspection of diverse transcriptome datasets revealed that each *NvPrdm14* paralog is overrepresented in at least one neural transcriptome (Supplementary Table 1)^44,73,74^, but by in situ hybridization (ISH), we obtained a clear expression pattern only for *NvPrdm14d*.

We found that *NvPrdm14d* is expressed in very few single ectodermal cells at early gastrula stage (Fig. 1B), before starting to be expressed in individual cells in the pharynx from mid-gastrula (Fig. 1C). The colorimetric in situ hybridizations did not allow to decide unambiguously whether the pharyngeal staining is located in the ectodermal, the endodermal or both parts of the pharynx. The strong expression in cells in the pharynx lasts until the mid-planula stage (Fig. 1F). From early planula stage, *NvPrdm14d* is expressed in scattered endodermal cells in the body wall (Fig. 1E) and from mid-planula, it is expressed in these scattered endodermal and some pharyngeal cells (Fig. 1G–I). In addition, *NvPrdm14d* is expressed in a small number of ectodermal cells during gastrula and planula stages (Fig. 1C–I). In the primary polyp, *NvPrdm14d* is expressed in some gastrodermal domains on the oral side (Fig. 1J). Overall, the expression pattern in scattered cells and the onset of endodermal *NvPrdm14d*-expression at the stage when the first neural progenitor cells (NPCs) appear in the *Nematostella* endoderm^40^ are consistent with a role for *NvPrdm14d* in endodermal neurogenesis.

### *NvPrdm14d* is expressed in a subset of endodermal NPCs and their progeny

**Fig. 2:**
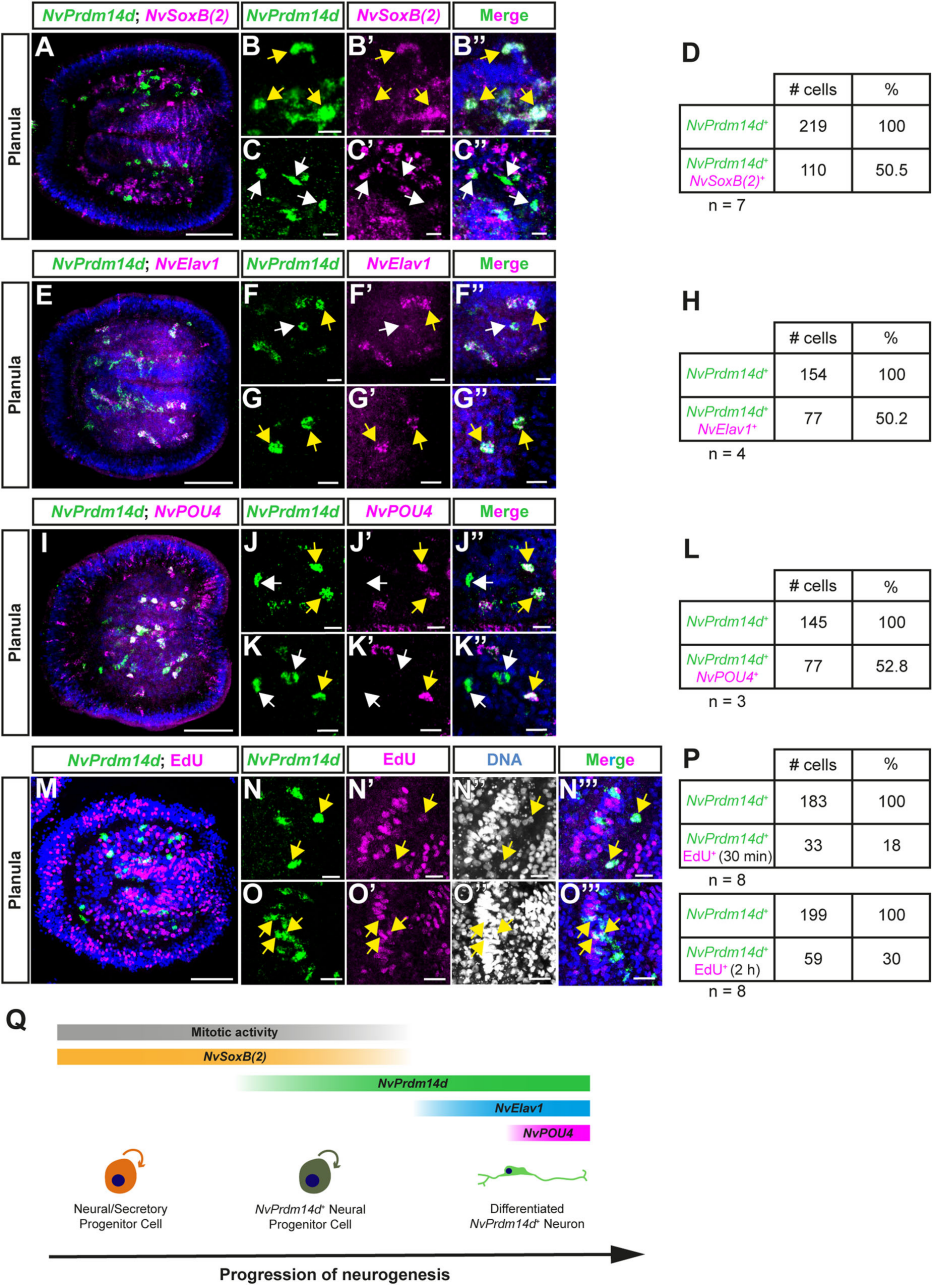
*NvPrdm14d* is expressed in a subset of endodermal neural/secretory progenitor cells as well as in their progeny. **A**–**C**, **E**–**G** and **I**–**K** Confocal images of double fluorescence in situ hybridization for *NvPrdm14d* (green) and different neural marker genes (magenta). **B**, **C** Enlargements of *NvPrdm14d*^+^ cells co-expressing (or not) *NvSoxB(2)*. **D** Quantification of *NvPrdm14d*^+^ cells co-expressing *NvSoxB(2)*: 15–76% (average 50.5%, *n* = 7) of *NvPrdm14d*^+^ cells co-express *NvSoxB(2)*. (F-G) Enlargements of *NvPrdm14d*^+^ cells co-expressing (or not) *NvElav1*. **H** Quantification of *NvPrdm14d*^+^ cells co-expressing *NvElav1*: 41–56% (average 50.2%, *n* = 4) of *NvPrdm14d*^+^ cells co-express *NvElav1*. **J**, **K** Enlargements of *NvPrdm14d*^+^ cells co-expressing (or not) *NvPOU4*. **L** Quantification of *NvPrdm14d*^+^ cells co-expressing *NvPOU4*: 50%–55% (average 52.8%, *n* = 3) of *NvPrdm14d*^+^ cells co-express *NvPOU4*. **M**–**O** Confocal images of fluorescence in situ hybridization for *NvPrdm14d* combined with the fluorescent labeling of proliferating cells by nuclear EdU staining, respectively shown in green and magenta. **N**–**O** Enlargements showing that some *NvPrdm14d*^+^ cells are co-labeled by EdU. DAPI is shown in white in (**N″, O″**) and in blue in the merged images (**N‴**, **O‴**). **P** Quantification of *NvPrdm14d*-expressing cells co-labeled by EdU. For 30 min pulse: 9–26% (average 18%, *n* = 8); for 2 h pulse: 22–33% (average 30%, *n* = 8) of *NvPrdm14d*^+^ cells are EdU positive. **Q** Schematics summarizing the temporal expression of *NvPrdm14d*. The expression starts in a subset of endodermal neural progenitor cells that are still dividing and express *NvSoxB(2)*. The expression of *NvPrdm14d* continues in neurons expressing differentiation markers such as *NvElav1* and *NvPOU4*. Yellow arrows indicate co-labeled cells, while white arrows indicate cells with a single label. Embryos are counterstained for DNA in blue, the oral pole is oriented to the right, scale bars: 50 µm for full embryos (**A**, **E**, **I**, **M**), 10 µm for enlargements (**B**, **C**, **F**, **G**, **J**, **K**, **N**, **O**).

To characterize the expression pattern of *NvPrdm14d* in more detail, we performed double fluorescence in situ hybridization (DFISH). The higher resolution afforded by DFISH and confocal imaging showed that the pharyngeal expression of *NvPrdm14d* is almost exclusively observed in the endodermal part of the pharynx (Fig. 2A, E, I, M). We then compared the expression pattern of *NvPrdm14d* with *NvSoxB(2)*, a gene that is expressed from blastula stage on in a population of progenitor cells that give rise to neurons and gland/secretory cells (neural/secretory progenitor cells, N/SPCs) in both the ectoderm and endoderm/gastrodermis in *Nematostella*^40,75,76^. At planula stage, quantification of confocal images showed that the number of cells expressing *NvSoxB(2)* was higher than that expressing *NvPrdm14d* (Fig. 2A). While on average half of the *NvPrdm14d*-expressing cells also express *NvSoxB(2)*, only a minority of *NvSoxB(2)*-expressing cells in the endoderm co-express *NvPrdm14d* (Fig. 2A–D). This suggests that *NvPrdm14d* is expressed in a subset of endodermal progenitor cells. Based on the much earlier onset of *NvSoxB(2)* expression^40,77^, we infer that the expression of *NvSoxB(2)* precedes that of *NvPrdm14d*.

Additionally, we compared the expression pattern of *NvPrdm14d* with the neuronal differentiation markers *NvElav1* and *NvPOU4*^39,44^ to determine whether the expression of endodermal *NvPrdm14d* is restricted to progenitor cells or lasts until neurons differentiate. At planula stage, we found that on average about 50% of the *NvPrdm14d*-expressing cells co-express *NvElav1* (Fig. 2E–H). We observed a similar proportion of *NvPrdm14d*^*+*^ cells expressing *NvPOU4* (Fig. 2I–L), consistent with the co-expression of *NvElav1* and *NvPOU4* in endodermal neurons^44^. This indicates that the expression of *NvPrdm14d* persists in differentiated endodermal neurons derived from the *NvPrdm14d*^+^ endodermal NPCs.

To test whether *NvPrdm14d* is expressed in proliferative cells, we combined FISH with EdU pulse labeling at planula stage. We found that after a 30 min EdU pulse on average 18%, and after a 2 h pulse, on average 30% of the *NvPrdm14d*^*+*^ cells are EdU positive (Fig. 2M–P), showing that at least some *NvPrdm14d*^*+*^ cells are dividing.

Altogether, our data suggest that the expression of *NvPrdm14d* starts in a subset of dividing *NvSoxB(2)*^+^ endodermal N/SPCs and persists in differentiated endodermal neurons after the expression of *NvSoxB(2)* ceased (Fig. 2Q). This interpretation is in agreement with the data generated in a recent single cell RNA-sequencing study (Supplementary Figure 1)^75^.

### *NvPrdm14d*^+^ cells differentiate mainly into ganglion neurons

To allow the visualization of the morphology of *NvPrdm14d*^+^ N/SPCs and their progeny, we generated a transgenic reporter line^78,79^ consisting of a gene encoding a membrane-tethered GFP under the control of a 5 kb sequence from the regulatory region of *NvPrdm14d*, referred to as *NvPrdm14d::GFP*.

**Fig. 3:**
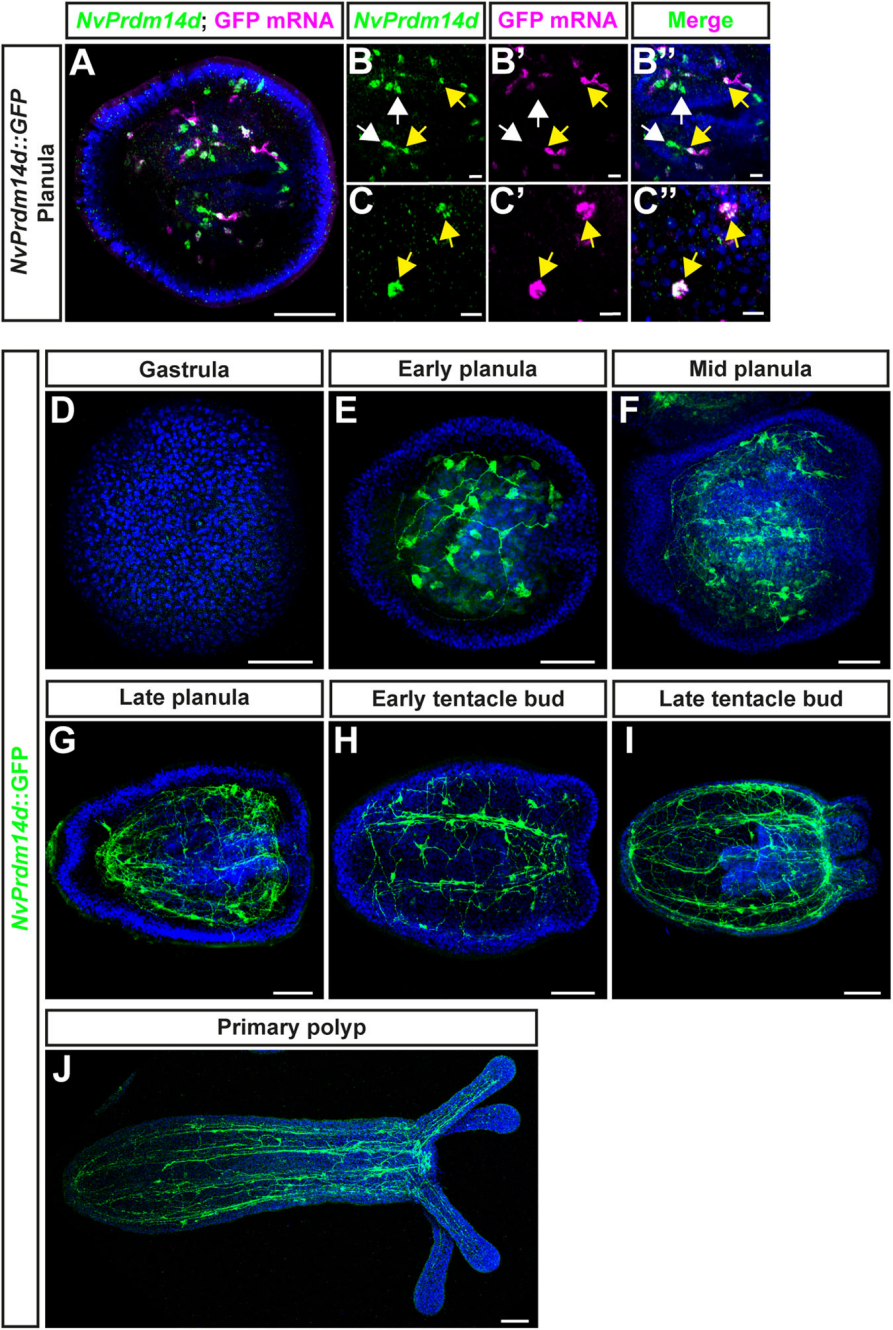
A transgenic reporter line for *NvPrdm14d* highlights a population of endodermal/gastrodermal neurons. **A**–**C** Confocal images of double fluorescence in situ hybridization for *NvPrdm14d* (green) and GFP mRNA (magenta) in *NvPrdm14d*::GFP transgenic embryos. **B**, **C** Enlargements of *NvPrdm14d*^+^ cells co-expressing (or not) GFP mRNA. Most GFP^+^ cells co-express *NvPrdm14d* (yellow arrows). However, several *NvPrdm14d*^*+*^ cells are GFP^-^, notably in the pharynx (white arrows). **D**–**J** Time course of confocal images of immunofluorescence staining for *NvPrdm14d*::GFP over developmental stages revealing neurons forming a subset of the endodermal/gastrodermal nerve net and of the neurites composing the longitudinal tracts. GFP is shown in green. Samples are counterstained for DNA in blue. In all pictures, the oral pole is oriented to the right. Scale bars: 50 µm for full embryos (**A**, **D**–**H**), 10 µm for enlargements (**B**, **C**).

DFISH for *NvPrdm14d* and GFP mRNAs in *NvPrdm14d::GFP* transgenic planulae showed that most *NvPrdm14d*^+^ cells co-express GFP mRNA and that all cells expressing *GFP* mRNA are *NvPrdm14d*^+^ (Fig. 3A–C). We found, however, that some *NvPrdm14d*^+^ cells do not co-express GFP mRNA, particularly in the pharynx (Fig. 3A–C).

**Fig. 4:**
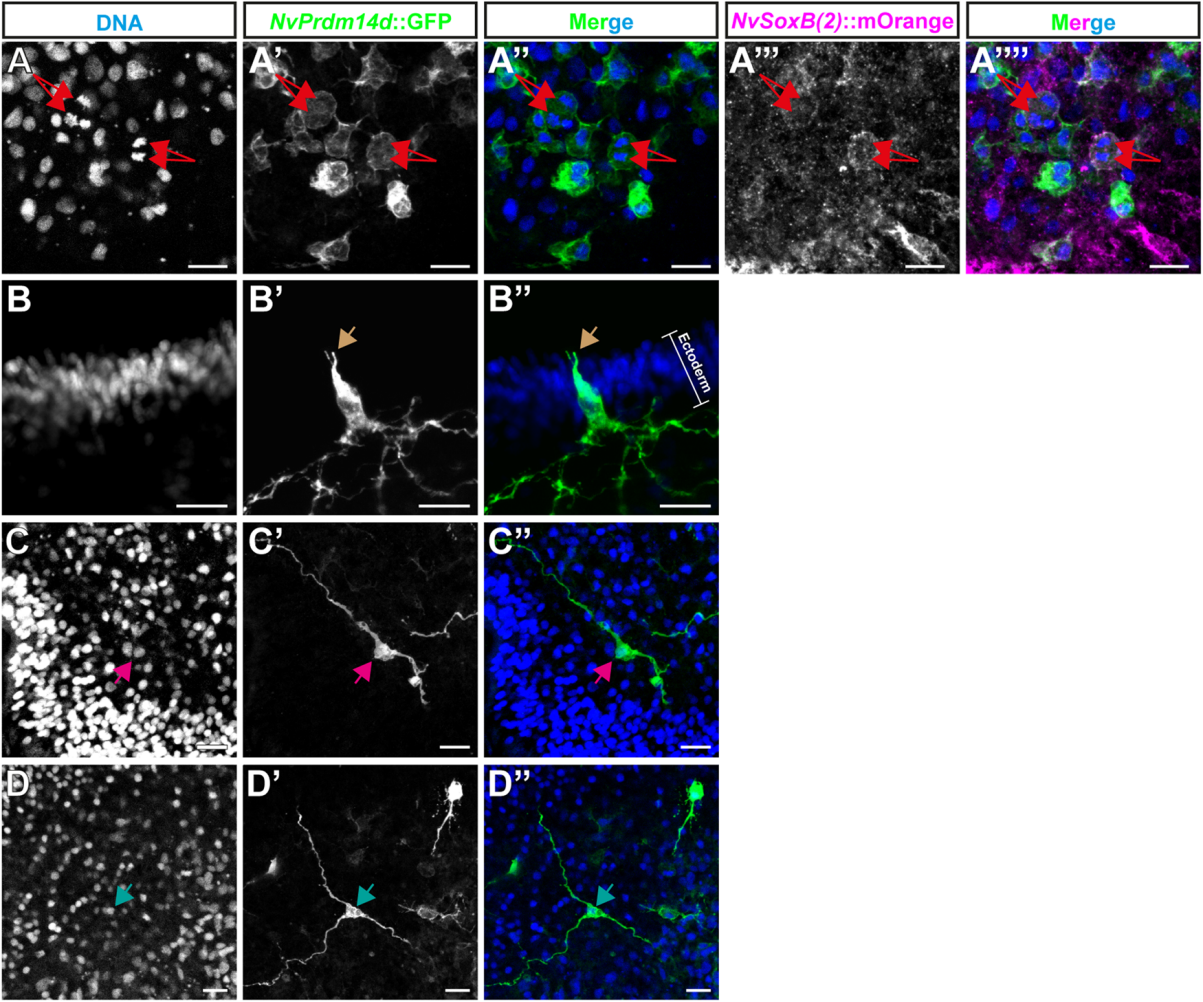
Cells expressing *NvPrdm14d*::GFP are mainly endodermal/gastrodermal ganglion neurons. Overview of the different types of cells labeled by *NvPrdm14d*::GFP, shown in green. **A** *NvPrdm14d:*:GFP labels dividing endodermal neural progenitor cells (NPCs) as these cells are co-labeled by *NvSoxB(2)*::mOrange, shown in magenta. Arrows indicate sister chromatids at anaphase. **B** Consistent with the expression pattern of *NvPrdm14d*, some rare ectodermal sensory neurons are labeled by *NvPrdm14d*::GFP in the central region of the oral-aboral axis. Arrows indicate the sensory cilium; the white bracket indicates the ectoderm. **C**, **D** The endodermal neurons labeled by *NvPrdm14d*::GFP are ganglion cells with a variable number of neurites, e.g. bi- and tripolar neurons as depicted here. No asymmetric distribution of these ganglion neurons was observed along the embryo axes. Arrows indicate neural somata. DAPI is shown in blue in the merged images. Scale bars: 10 µm.

The analysis of *NvPrdm14d*::GFP-expressing animals identified GFP^+^ neurons in the endoderm (Fig. 3D–J). Expression of the transgene can be visualized from early planula in cells exhibiting neurite projections, however some GFP^+^ cells lack neurites at this stage (Figs. 3E, 4A). During larval development, an increasing number of neurons are labeled by GFP, and neurites progressively form longitudinal tracts along the mesenteries (infoldings that are mainly derived from the body wall endoderm) as well as the endodermal nerve net (Fig. 3F–I). Additionally, we detect GFP^+^ neurites in the tentacles of the primary polyp (Fig. 3J). Compared to previously described transgenic reporter lines for *NvSoxB(2)* and *NvElav1*^39,40^, the *NvPrdm14d* reporter labels a smaller number of cells. We did not observe cells with the morphology of gland/secretory cells.

Next, we looked more closely at individual GFP^+^ cells, and we noticed that some of the cells lacking projections in early planulae, are dividing (Fig. 4A–A″). We crossed the *NvPrdm14d::GFP* line with the previously described *NvSoxB(2)::mOrange* line to obtain double transgenics^40^. In such animals, the GFP^+^ dividing cells are also mOrange^+^ (Fig. 4A‴–A⁗), confirming that the reporter line allows the visualization of *NvPrdm14d*^+^ NPCs and their progeny.

In late planulae, we could observe some rare ectodermal neurons labeled by GFP. Only few of the screened larvae exhibited such cells (12%, *n* = 42) and only few ectodermal cells were found in individual larvae (1–3 cells). These cells are likely derived from the few ectodermal cells expressing *NvPrdm14d* as revealed by ISH (Fig. 1). These GFP^+^ ectodermal neurons are found in the central region along the oral-aboral axis, are elongated along the apical-basal axis of the ectoderm and display an apical cilium (Fig. 4B–B″). The morphological characteristics of these cells suggest that they are sensory neurons.

By contrast, most GFP^+^ neurons in planula larvae have their soma located at a basal position within the endodermal epithelium, suggesting that they are ganglion neurons (Fig. 4C, D″). These endodermal neurons exhibit a variable number of neurites, hence can be identified as bipolar and tripolar neurons. However, no specific spatial distribution of these neurons was detected.

These results show that the *NvPrdm14d* reporter labels a subset of the endodermal NPCs as well as their progeny, which mainly consists of ganglion neurons.

### *NvPrdm14d*^+^ cells generate a subset of endodermal/gastrodermal neurons

To further investigate the identity of the endodermal/gastrodermal neurons generated by *NvPrdm14d*^+^ NPCs, we crossed the transgenic line with other reporter lines that have been characterized previously.

**Fig. 5:**
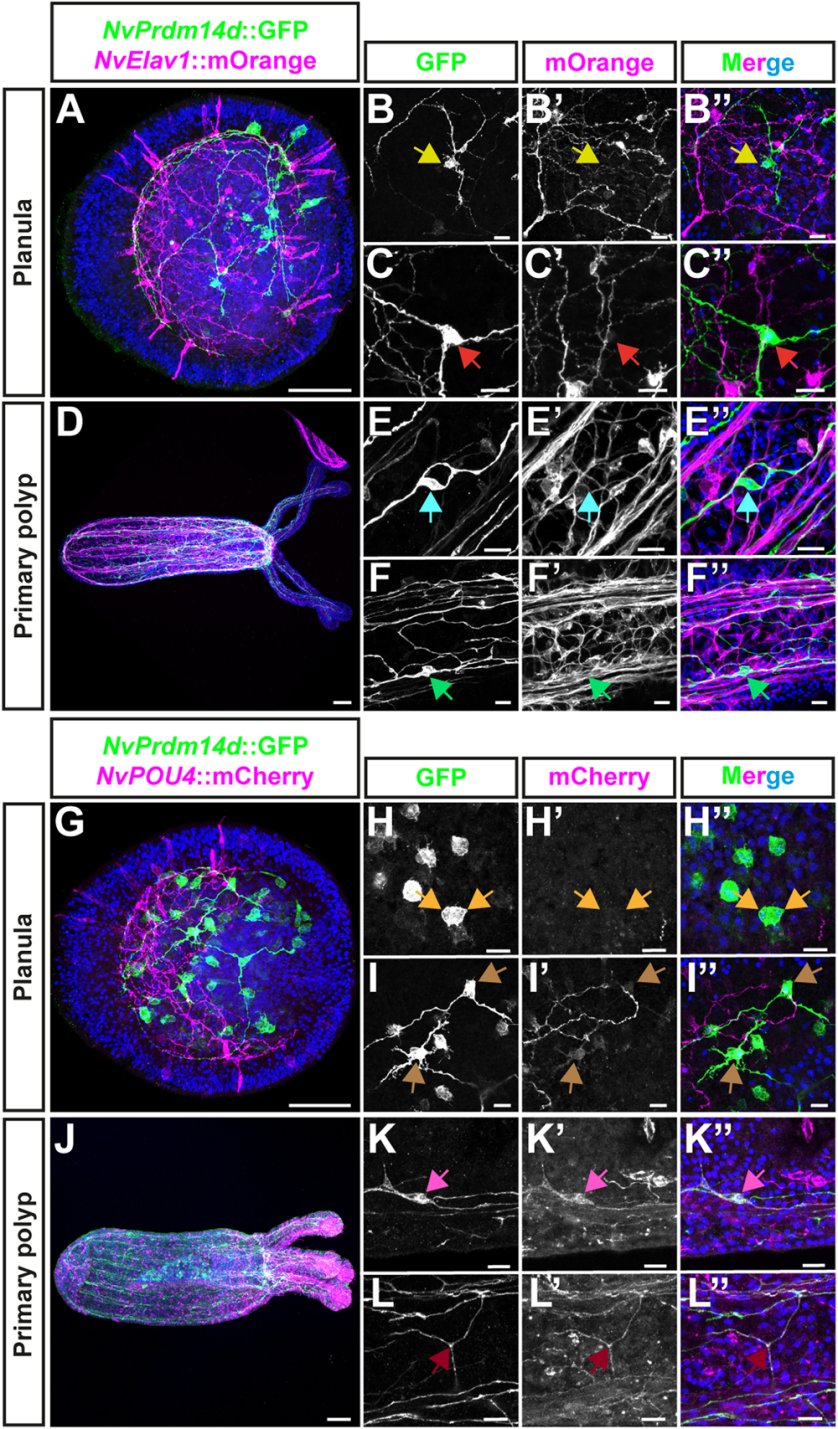
*NvPrdm14d*::GFP highlights a subset of endodermal/gastrodermal *NvPOU4*::mCherry^+^ neurons but not of *NvElav1*::mOrange^+^ neurons. **A**–**F** Confocal images of immunofluorescence staining for *NvPrdm14d*::GFP and *NvElav1*::mOrange, respectively shown in green and magenta. **A**–**C** Planula stage. **D**–**F** Primary polyp stage. **B**, **C**, **E**, **F** Enlargements showing that endodermal/gastrodermal neurons expressing *NvPrdm14d*::GFP do not express *NvElav1*::mOrange. Arrows indicate the soma of *NvPrdm14d*::GFP^+^ neurons that do not express *NvElav1*::mOrange. **G**–**L** Confocal images of immunofluorescence staining for *NvPrdm14d*::GFP and *NvPOU4*::mCherry, respectively shown in green and magenta. **G**–**I** Planula stage. **J**–**L** Primary polyp stage. **H** Enlargements showing dividing NPCs expressing *NvPrdm14d*::GFP but not *NvPOU4*::mCherry, consistent with the role of *NvPOU4* in terminal differentiation. Arrows indicate chromosomes at anaphase in *NvPrdm14d*::GFP^+^ NPCs that do not express *NvPOU4*::mCherry. **I,** **K**, **L** Enlargements showing that differentiated neurons expressing *NvPrdm14d*::GFP also express *NvPOU4*::mCherry. Arrows indicate neurons or neurites co-expressing *NvPrdm14d*::GFP and *NvPOU4*::mCherry. Note that the neurites with bright mCherry signal in (**I**′) do not emerge from the soma labeled with the brown arrow. Embryos are counterstained for DNA in blue, the oral pole is oriented to the right, scale bars: 50 µm in (**A**, **D**, **G,** **J**) and 10 µm in (**B**, **C**, **E**, **F**, **H**, **I**, **K**, **L**).

The *NvElav1::mOrange* reporter line has been shown to label a large population of differentiated neurons in both the ectoderm/epidermis and the endoderm/gastrodermis, but not cnidocytes^39^. Though many *NvPrdm14d*::GFP^+^ neurons and neurites are in close proximity of *NvElav1*::mOrange^+^ neurons in primary polyps, we did not detect any co-expression of GFP and mOrange in both planula larvae and primary polyps (Fig. 5A–F″). This was unexpected since *NvPrdm14d* and *NvElav1* are partially co-expressed in the DFISH experiment (Fig. 2E–H) and in single cell RNA-sequencing data at developmental stages (Supplementary Fig. 1)^75^. Neither of the transgenic reporter lines matches the expression of the endogenous gene perfectly^39^ (Fig. 3A–C) and we assume that neurons that co-express the *NvPrdm14d* and *NvElav1* transcripts are among those that are not identified with one or both of the transgenic lines. Together, these data show that the *NvElav1*::mOrange and *NvPrdm14d*::GFP lines label two distinct populations of neurons.

Next, we crossed the *NvPrdm14d::GFP* line with the *NvPOU4::mCherry* reporter line that has been shown to label ectodermal and gastrodermal neurons, as well as cnidocytes in *Nematostella*^44^. At planula stage, we observed partial co-expression of GFP and mCherry (Fig. 5G–I″), which matches our observation that *NvPrdm14d* and *NvPOU4* transcripts are partially co-expressed (Fig. 2I–L). Among the cells co-labeled by both transgenes, we did not observe any of the *NvPrdm14d*::GFP^+^ cells that lack neurites or divide (Fig. 5H–H″). However, *NvPrdm14d*::GFP^+^ cells exhibiting neurites always co-express *NvPOU4*::mCherry (Fig. 5I–I″), although frequently at comparably low levels. This is in line with the putative role of *NvPOU4* in terminal differentiation of neurons^44^. Furthermore, we noted that the *NvPrdm14d*::GFP^+^ gastrodermal neurons co-express the *NvPOU4*::mCherry reporter in the primary polyp, but that the majority of *NvPOU4*::mCherry^+^ neurons are *NvPrdm14d*::GFP^–^ (Fig. 5J–L″).

The analysis of these double transgenic animals reveals that the *NvPrdm14d*::GFP and *NvPOU4*::mCherry reporters are co-expressed in a population of post-mitotic neurons and that the *NvPrdm14d*^+^ NPCs likely generate only a small subset of gastrodermal neurons.

### The *NvPrdm14d*::*GFP* transgene labels neurons close to retractor muscles

A role for *Prdm14* in neurogenesis has been described in zebrafish where it is involved in the axon outgrowth of primary motoneurons^54^. We therefore decided to explore the possibility that *NvPrdm14d* might be involved in the development of motoneurons in *Nematostella*.

**Fig. 6:**
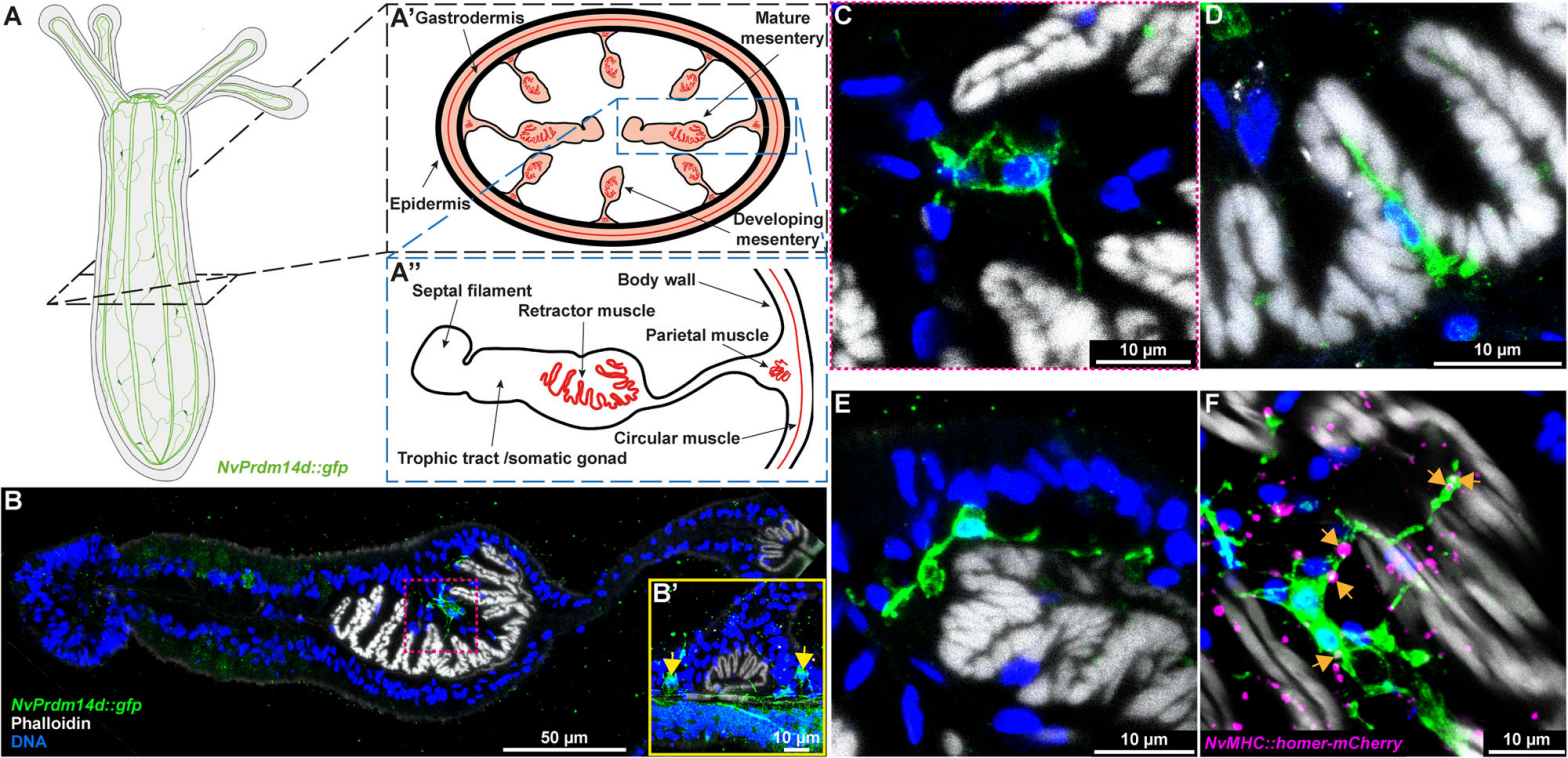
*NvPrdm14d*::GFP^+^ neurons are in the close vicinity of retractor muscles. **A** Schematic illustrating the experimental sectioning performed to observe *NvPrdm14d*::GFP^+^ neurons in the vicinity of retractor muscles. The different structures observed on sections and in mesenteries are described in **(A**′–**A**″). **B**–**F** Confocal images of immunofluorescence staining for *NvPrdm14d*::GFP in mesenteries. GFP is shown in green and muscles in white. (**B**’) Enlargement of a parietal muscle showing a concentration of neurites on both sides (arrows), corresponding to the longitudinal tracts. **C**–**F** Enlargements of different neurons expressing the *NvPrdm14d*::GFP in the vicinity of the retractor muscles. **C** Enlargement corresponding to the dashed line square shown in (**B**). **F** *NvPrdm14d*::GFP-expressing neurons project neurites toward retractor muscles and are in contact with putative neuromuscular synaptic junctions. The putative neuromuscular synaptic junctions are revealed by a fusion protein homer-mCherry under the control of the retractor muscle-specific promoter *NvMyHC1*, shown in magenta. Arrows indicate putative neuromuscular post-synapses in contact with *NvPrdm14d*::GFP^+^ neurons/neurites. Scale bars: 50 µm in (**B**), 10 µm in (**B**′–**F**).

To this end, we crossed the *NvPrdm14d::GFP* reporter line with the *NvMyHC1::mCherry* reporter line, specifically labeling retractor muscles in the mesenteries and tentacle muscles through the expression of mCherry under the *Myosin Heavy Chain-ST* promoter^78^. In primary polyps, we observed that some of the *NvPrdm14d*::GFP^+^ neurons are near the retractor muscles (Supplementary Figure 2A–C), which are located on gastrodermal infoldings of the body wall called mesenteries (Fig. 6A–A″). However, the developing retractor muscles are close to the parietal muscle and the longitudinal neurite tracts of the body wall at this stage, confounding the analysis of a potential association with *NvPrdm14d*::GFP^+^ neurons.

Therefore, we decided to image 2- to 4-month-old polyps with well-developed mesenteries (Fig. 6). The analysis of mesentery cross-sections stained with phalloidin (to visualize F-actin) revealed a concentration of *NvPrdm14d*::GFP^+^ neurites, likely corresponding to the longitudinal neurite tracts, as well as some cell bodies located on each side of the parietal muscles (Fig. 6B′). We also observed some *NvPrdm14d*::GFP^+^ neurons within the mesenteries in close vicinity of retractor muscles (Fig. 6B–E). We did not find *NvPrdm14d*::GFP^+^ neurons in any other part of the mesenteries. Interestingly, these neurons were observed at diverse locations around the retractor muscles, e.g. close to or within the gastrodermal epithelium (Fig. 6C, E), or in the folds of the retractor muscles (Fig. 6D). All these neurons appear to project neurites either towards the contractile filaments of the retractor muscles or along them, suggesting that they might establish synaptic connections with these muscles.

To address this in more detail, we generated a *NvMyHC1::homer-mCherry* line expressing the putative post-synaptic protein homer, fused to mCherry, under the control of the retractor muscle-specific *NvMyHC1* promoter, presumably allowing the visualization of post-synaptic sites in the retractor muscles. Indeed, the homer-mCherry fusion protein is detected in puncta arranged in longitudinal tracts in the body column and tentacles of primary polyps (Supplementary Fig. 3A–E), consistent with the expression of the mCherry protein labeling retractor and tentacle muscles in *NvMyHC1::mCherry* primary polyps [Supplementary Fig. 3L–P^78^]. Following cross-sectioning of *NvMyHC1*::homer-mCherry^+^ polyps, we observed that puncta are found close to the contractile filaments of the retractor muscles as revealed by phalloidin staining, both in mature and in developing mesenteries (Supplementary Figure 3H–K). We did not observe such puncta in *NvMyHC1*::mCherry polyps (Supplementary Figure 3H–K & S–V). These findings are in agreement with the detection of neuromuscular synapses in the *Nematostella* retractor muscle by electron microscopy^80^.

We then analyzed cross-sections of *NvPrdm14d::GFP*; *NvMyHC1::homer-mCherry* double transgenic animals. We observed that somata and neurites of the *NvPrdm14d*::GFP^+^ neurons located in the vicinity of retractor muscles are in close contact with *NvMyHC1*::homer-mCherry^+^ puncta (Fig. 6F). This observation supports the hypothesis that *NvPrdm14d*::GFP^+^ neurons establish synaptic connections with retractor muscles.

We did not observe any *NvPrdm14d*::GFP^+^ neurons or neurites in the mesentery tissue connecting the body wall and the retractor muscles (Fig. 6B). When we analyzed cross-sections of *NvPrdm14d::GFP*; *NvElav1::mOrange* double transgenic polyps, we could not detect *NvElav1*::mOrange^+^ neurons in the mesentery tissue between the body wall and the retractor muscle (Supplementary Fig. 2D). The existence of an additional population of neurons, not labeled by the two transgenes, is a possible explanation for this observation.

Together, these data reveal that the *NvPrdm14d*::GFP reporter highlights a population of previously undescribed potential motoneurons in the vicinity of retractor muscles.

### *NvPrdm14d*^+^ cells do not originate in the *NvFoxA*^+^ ectodermal pharynx

Since the expression pattern of *NvPrdm14d* is dynamic during embryonic development with a strong expression in the pharynx before being mainly expressed in scattered endodermal cells (Fig. 1), we wanted to take advantage of the *NvPrdm14d::GFP* transgenic line to confirm the endodermal origin of the *NvPrdm14d*^+^ cells. Indeed, the part of the pharynx lining the oral opening has an ectodermal origin, hence it is possible that the *NvPrdm14d*^+^ NPCs originate in this domain before migrating to the endoderm at planula stage.

**Fig. 7:**
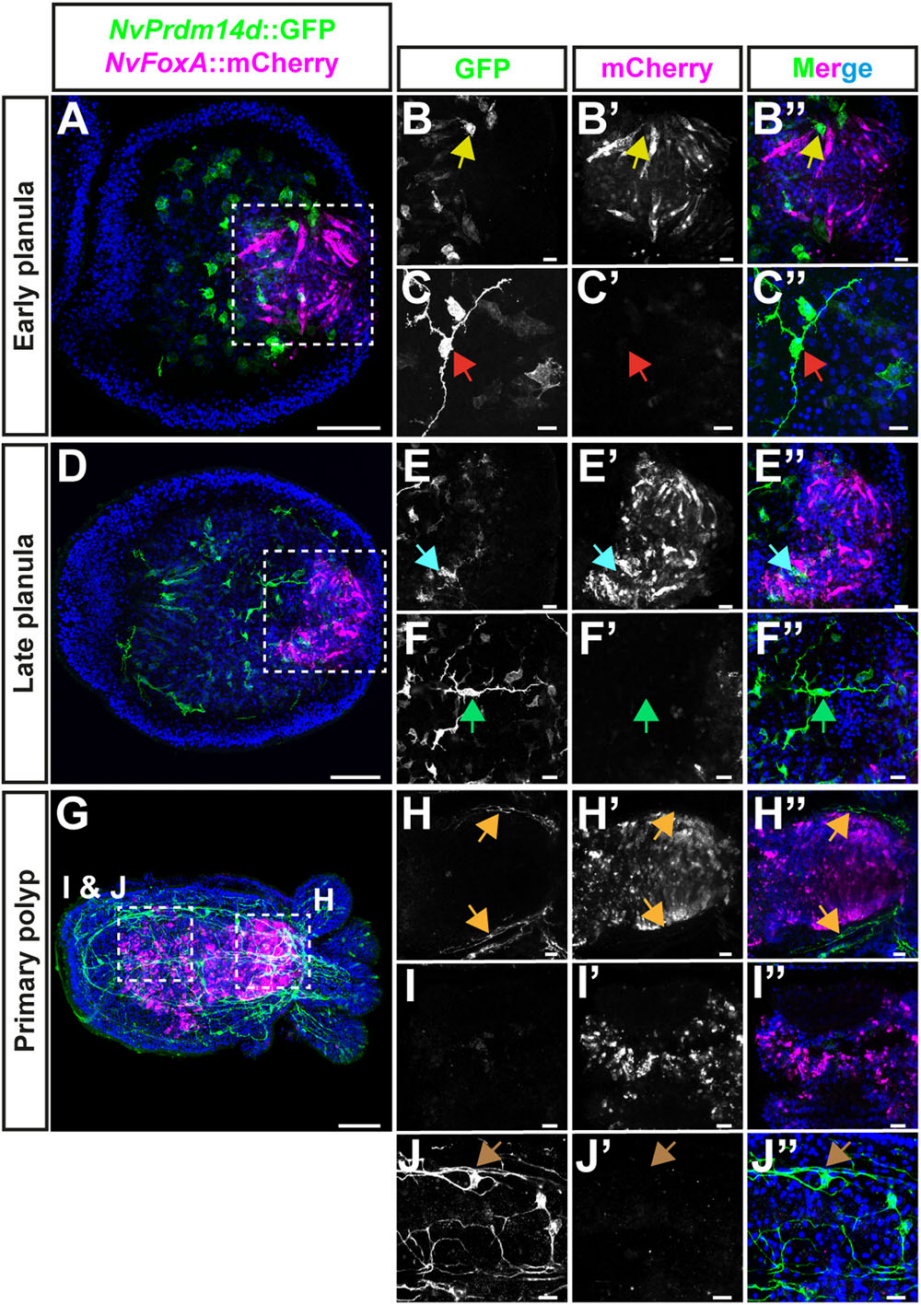
*NvPrdm14d*::GFP^+^ neurons do not originate from *NvFoxA*::mOrange2-expressing cells of the ectodermal pharynx. **A**–**J** Confocal images of immunofluorescence staining for *NvPrdm14d*::GFP and *NvFoxA*::mOrange2, respectively shown in green and magenta. The promoter of *NvFoxA* drives gene expression in the ectodermal pharynx, specifically. **A**–**F** Planula stages. **G**–**J** Primary polyp stage. **B**, **E**, **H** Enlargements showing that ectodermal pharyngeal cells express *NvFoxA*::mOrange2 but not *NvPrdm14d*::GFP. Arrows indicate that *NvPrdm14d*::GFP^+^ neurons are negative for *NvFoxA*::mOrange2. **I** Enlargements showing ectodermal pharyngeal cells in the distal part of the mesenteries of primary polyps. Those cells express *NvFoxA*::mOrange2 but not *NvPrdm14d*::GFP. **C**, **F**, **J** Enlargements showing that endodermal/gastrodermal neurons expressing *NvPrdm14d*::GFP, do not express *NvFoxA*::mOrange2. Arrows indicate the soma of some *NvPrdm14d*::GFP^+^ neurons showing an absence of *NvFoxA*::mOrange2 expression. Embryos are counterstained for DNA in blue, the oral pole is oriented to the right, scale bars: 50 µm in (**A**, **D**, **G**) and 10 µm in **(B**, **C**, **E**, **F**, **H**, **J**).

To address this possibility, we used a transgenic reporter line expressing mOrange2 under the control of a 5.9 kb long *NvFoxA* promoter^25,26,81^. At early planula stage, this fluorescent reporter is exclusively expressed in the ectodermal part of the pharynx (Fig. 7A), though we cannot exclude that some cells of the ectodermal pharynx do not express the transgene. After crossing to the *NvPrdm14d::GFP* line, we observed that few *NvPrdm14d*::GFP^+^ cells are neighboring *NvFoxA*::mOrange2^+^ cells, but none of them express both reporters (Fig. 7B–B”). By contrast, most of *NvPrdm14d*::GFP^+^ cells in the pharynx are distant from *NvFoxA*::mOrange2^+^ cells. Moreover, none of the *NvPrdm14d*::GFP^+^ differentiated neurons express *NvFoxA*::mOrange2 (Fig. 7C–C″). Similar observations were made in late planula larvae that exhibit a broader expression of *NvFoxA*::mOrange2 in the ectodermal pharynx with a higher number of *NvFoxA*::mOrange2^+^ cells (Fig. 7D–F″). In the primary polyp, *NvFoxA*::mOrange2^+^ cells are found in the pharynx and in the distal part of the mesenteries where they form structures called septal filaments (Fig. 7G), consistent with a previous report^26^. Similar to our description in planula larvae, we did not detect any *NvFoxA*::mOrange2^+^ cells in the ectoderm-derived part of the pharynx or the septal filaments that co-express *NvPrdm14d*::GFP (Fig. 7H–I″). At this stage, none of the *NvPrdm14d*::GFP^+^ differentiated neurons co-express *NvFoxA*::mOrange2 either (Fig. 7J–J″).

Taken together, these observations support the conclusion that endodermal/gastrodermal *NvPrdm14d*^+^ cells mainly originate from the endoderm, including the endodermal region of the pharynx, and not from ectodermal pharyngeal cells.

### Transcriptome analysis of *NvPrdm14d*::GFP^+^ cells

**Fig. 8:**
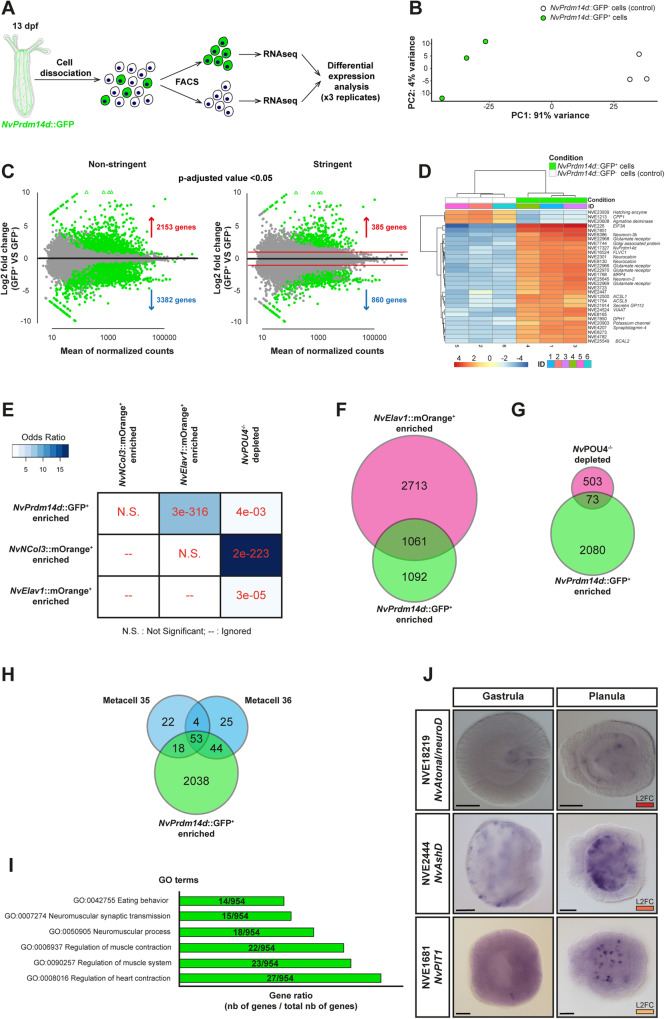
Transcriptomic analysis of *NvPrdm14d*::GFP^+^ cells. **A** Schematic illustrating the experimental design. **B** Principal component analysis (PCA) of gene counts after variance stabilizing transformation (VST). **C** MA-plots of enriched and depleted genes between *NvPrdm14d*::GFP^+^ cells and *NvPrdm14d*::GFP^-^ controls. Genes that exhibit differential expression (*p*-adjusted value < 0.05) in green. The plot on the left (non-stringent) shows the number of differentially expressed genes without applying log2 fold change (LFC) threshold. “Stringent” shows the number of differentially expressed genes when the LFC threshold of 1 was directly included in the statistical analysis. **D** Gene clustering matrix showing a subset of the most highly variable genes explaining the difference between *NvPrdm14d*::GFP^+^ and *NvPrdm14d*::GFP^-^ cells. Note that *NvPrdm14d* appears there. **E** Comparison of the overlap between non-stringent enriched genes in *NvPrdm14d*::GFP^+^ cells with those enriched in *NvElav1*::mOrange^+^ and *NvNCol3*::mOrange^+^ cells; as well as with those depleted in *NvPOU4* mutants. The comparison is done using the GeneOverlap R package and Fisher’s exact test. The intensity of the blue color indicates the odds ratio (values greater than 1 imply association), and numerical values represent the *p*-value (smaller values indicate a more significant overlap). **F** Venn diagram comparing the number of non-stringent genes enriched in *NvPrdm14d*::GFP^+^ cells with those enriched in *NvElav1*::mOrange^+^ cells. **G** Venn diagram comparing the number of non-stringent genes enriched in *NvPrdm14d*::GFP^+^ cells with those depleted in *NvPOU4* mutants. **H** Venn diagram comparing the number of non-stringent genes enriched in *NvPrdm14d*::GFP^+^ cells with those characterizing the neuronal metacells 35 and 36 from the single cell transcriptome of whole *Nematostella* by ref. ^74^. **I** GO terms, associated with motoneuron function, found among non-stringent genes enriched in *NvPrdm14d*::GFP^+^ cells. The gene ratio shows the number of enriched genes associated with these terms in *NvPrdm14d*::GFP^+^ cells, compared to the total number of enriched genes in *NvPrdm14d*::GFP^+^ cells that have been annotated with a GO term. **J** In situ hybridization of genes enriched in the *NvPrdm14d*::GFP^+^ cells. L2FC: Log2 fold change, the strength of the red color indicates the level of enrichment as shown in (**D**). Scale bars: 50 µm.

To gain further insights into the characteristics of neurons derived from *NvPrdm14d*^+^ NPCs, we separated *NvPrdm14d*::GFP^+^ and *NvPrdm14d*::GFP^−^ (control) cells using fluorescence-activated cell sorting (FACS) at primary polyp stage and performed RNA sequencing on both cell populations in triplicates (Fig. 8A, B).

In the *NvPrdm14d*::GFP^+^ cells, a total of 5535 genes are differentially expressed, including 2,153 enriched and 3382 depleted genes (“non-stringent list”, *p* < 0.05, Fig. 8C left). Additionally, we generated a “stringent list” by removing all differentially expressed genes with a Log2Fold Change between −1 and 1. This allowed the identification of 1245 differentially expressed genes, including 285 enriched and 860 depleted genes (*p* < 0.05, Fig. 8C right). Within these lists, we found *NvPrdm14d* as one of the most differentially expressed genes supporting the notion that *NvPrdm14d* remains expressed in at least some of the GFP^+^ neurons at least until primary polyp stage (Fig. 8D).

First, we looked at the non-stringent list and sought for genes that would help characterizing the neural features of *NvPrdm14d*::GFP^+^ cells. We found many genes annotated as receptors for glutamate, neuropeptide FF, pyroglutamylated RFamide, acetylcholine and dopamine (Supplementary Data 1). We also found some genes annotated as receptors for adrenaline and glycine. Additionally, we observed an enrichment in calcium-, sodium- and potassium-gated channels. Moreover, we identified several genes encoding putative pre-synaptic proteins, such as synaptotagmins (Supplementary Data 1).

Then, we analyzed whether target genes and/or genes encoding functional or physical interactors of Prdm14 known from other species, were found among the upregulated genes in *NvPrdm14d*::GFP^+^ cells. We found that *NvIslet* (NVE10444), a target of Prdm14 in zebrafish motoneurons, is upregulated in *NvPrdm14*::GFP^+^ cells (stringent list, Supplementary Data 1). In addition, we found the Prdm14 partners *NvCBFA2T* (NVE5561) and *NvEed-A* (NVE21521, part of PRC2) in the non-stringent list, indicating that interaction of Prdm14 and PRC2 might occur in *NvPrdm14d*::GFP^+^ neurons (Supplementary Data 1).

Next, we compared the *NvPrdm14d*::GFP^+^ transcriptome with previously generated transcriptomes, *i.e*. for *NvNCol3*::mOrange2^+^ and *NvElav1*::mOrange^+^ cells^44,73^. All three transcriptomes were generated from primary polyps at 13 dpf (days post fertilization). For each comparison, we used the GeneOverlap R package to test whether the gene overlap is random or not. This test provides the odds ratio (strength of association) and its p-value (significance of the association). An odds ratio equal or below 1 means no association, while the association is stronger with a higher ratio. We found a small overlap between genes enriched in *NvPrdm14d*::GFP^+^ cells (~14.3%) and those depleted in *NvNCol3*::mOrange2^+^ cells, however the odds ratio indicates that this overlap is not significant (Fig. 8E).

By contrast, we found that about 50% of the genes enriched in *NvPrdm14d*::GFP^+^ cells are also enriched in *NvElav1*::mOrange^+^ cells, and this overlap is significant (Fig. 8E, F). We also found *NvElav1* among the enriched genes (stringent list, Supplementary Data 1). This is in line with the partial co-expression of *NvPrdm14d* and *NvElav1* as shown by our DFISH experiment (Fig. 2E–H) and in single cell data (Supplementary Figure 1)^75^, and it suggests that the *NvElav1::mOrange* transgenic reporter line does not label the entirety of *NvElav1*^+^ cells.

As we observed co-expression of *NvPrdm14d* and *NvPOU4* (Fig. 2I–L), and of the *NvPrdm14d*::GFP and *NvPOU4*::mCherry reporters (Fig. 5G–I″), we compared our transcriptome with the one of *NvPOU4* mutants, referred to as *NvPOU4*^−/−^^44^. We found a small, but significant, overlap between genes enriched in *NvPrdm14d*::GFP^+^ cells (~3.4%) and those depleted in *NvPOU4*^-/-^ (Fig. 8E, G). Therefore, *NvPOU4* might regulate aspects of the terminal differentiation of *NvPrdm14d* ^+^ neurons.

Furthermore, we took advantage of the whole-animal single cell transcriptome atlas of *Nematostella* and searched for neuronal metacells expressing *NvPrdm14d*^74^. We found two metacells (35 & 36) expressing *NvPrdm14d* and they respectively share 73.5% and 77.2% of their defining genes with genes enriched in the *NvPrdm14d*::GFP^+^ transcriptome (Fig. 8H, Supplementary Data 2). Moreover, these two metacells share about 50% of their defining genes (Fig. 8H). However, we did not find such an overlap in gene expression when we analyzed genes enriched in the *NvPrdm14d*::GFP^+^ transcriptome within all the neuronal metacells, confirming the specific expression of *NvPrdm14d* in the metacells 35 and 36. A second single cell RNA-sequencing study also identified *NvPrdm14d* expression in two groups of cells in adult tissue, annotated as neural progenitor cells and gastrodermal neurons, respectively^75^ (Supplementary Fig. 1). Altogether, these data support the hypothesis that *NvPrdm14d* is expressed in, at least, two populations of cells belonging to the neural lineage.

With the aim to understand unique characteristics of *NvPrdm14d*::GFP^+^ neurons, we next looked at genes that are enriched in these neurons, but not in *NvElav1*::mOrange^+^ cells. This revealed an enrichment in GO terms associated with chromosome and chromatin organization, as well as cell cycle regulation (Supplementary Data 3). While this is not informative about the potential role played by *NvPrdm14d*::GFP^+^ neurons, it is consistent with the observation that *NvPrdm14d*-expressing cells include NPCs, whereas *NvElav1* is exclusively expressed in post-mitotic neurons. We then specifically searched for GO terms associated with motoneuron functions as *NvPrdm14d*::GFP^+^ neurons potentially establish synaptic connections with retractor muscles. We found six GO terms related to neuromuscular functions, however they are not the most enriched terms (Fig. 8I). Thus, the GO term analysis does not provide unambiguous support for a potential motoneuron identity among the *NvPrdm14d*::GFP^+^ neurons.

Finally, we focused on transcription factors enriched in *NvPrdm14d*::GFP^+^ cells and we checked their expression pattern in the literature, when existing. We found that most of the genes whose expression pattern is available, are expressed in the endoderm. These genes are *NvDmrt-b* [NVE6455^43,82^], *NvGATA* [NVE8199^25,26^], *NvGCM* [NVE12024^31,83^], *NvIslet* [NVE10444^26^], *NvNkx2*.2D [NVE10557^26^], *NvPOU4* [NVE5471^44^], *NvNkx3* [NVE18255^26,84^], *NvTbx20.3* [NVE24169^26^] and *NvSix1/2a* [NVE9850^26^]. Although some of these genes are additionally expressed in the ectoderm, we noticed that expression of *NvGCM*, *NvNkx2.2D*, *NvTbx20.3* and *NvSix1/2a* appears to be restricted to the endoderm, suggesting a role for these genes specifically in endodermal neurogenesis. ISH for some of the previously uncharacterized transcription factors enriched in the *NvPrdm14d*::GFP^+^ cells showed that *NvAtonal/neuroD* (NVE18219), *NvAshD* (NVE2444) and *NvPIT1* (NVE1681) are mainly expressed in scattered endodermal cells at planula stage, although they display expression in some ectodermal cells at gastrula stage (Fig. 8J). Interestingly, *NvAtonal/neuroD* was not found in genes enriched in *NvElav1*::mOrange^+^ cells, indicating that this gene might be exclusively expressed in the same lineage as *NvPrdm14d*. This gene is expressed in the pharynx of gastrula larvae, and then in scattered endodermal cells in planula larvae, though in fewer cells than *NvPrdm14d* (Supplementary Figure 4), suggesting that it might represent a subpopulation of *NvPrdm14d*::GFP^+^ neurons.

Taken together, the *NvPrdm14d*::GFP^+^ transcriptome analysis showed that *NvPrdm14d*::GFP^+^ neurons likely are a subpopulation of the *NvPOU4* endodermal/gastrodermal nervous system, that they overlap partially with *NvElav1*^+^ neurons and that they include putative motoneuron-like cells.

### Regulation of neural gene expression by *NvPrdm14d*

**Fig. 9:**
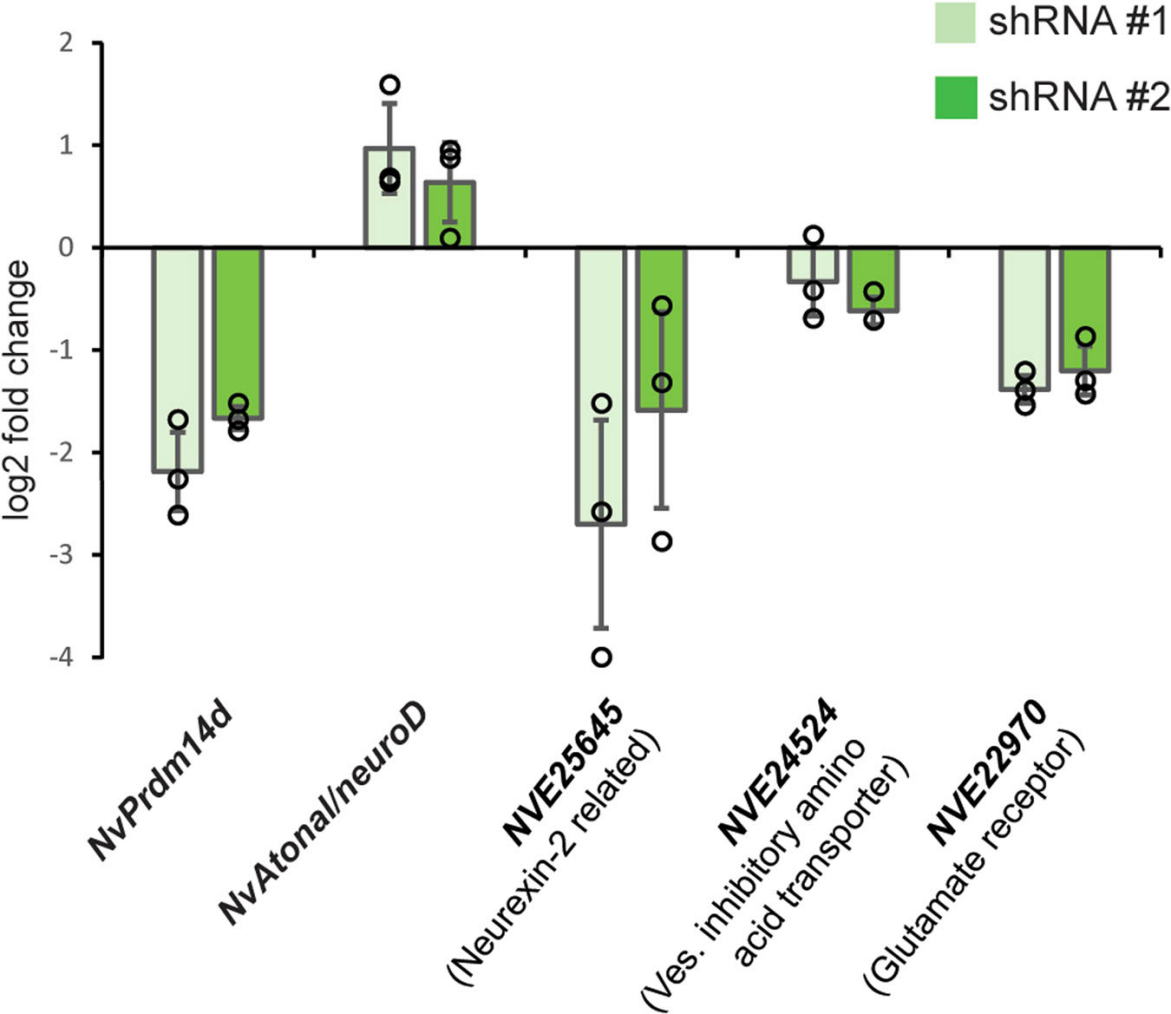
Regulation of gene expression by *NvPrdm14d*. qRT-PCR on selected neural genes upregulated in *NvPrdm14d*::GFP^+^, but not in *NvElav1*::mOrange^+^ cells, at early planula stage after shRNA injection. The graph shows the mean ± SD of the log2 fold change in expression between control shRNA and either *NvPrdm14d* shRNA #1 (light green) or #2 (green) across three independent biological replicates. The values for the individual replicates are shown as circles. Source data are provided as Source Data file.

To determine whether genes upregulated in *NvPrdm14d*::GFP^+^ cells are regulated by *NvPrdm14d*, we performed knockdown experiments by injection of shRNAs^85^. We designed two shRNAs that reduced *NvPrdm14d* transcript levels to 18.6% (log2 fold change −2.43) and 30.5% (log2 fold change −1.71), respectively, compared to animals injected with a control shRNA targeting GFP (Fig. 9). We selected four genes upregulated in *NvPrdm14d*::GFP^+^, but not in *NvElav1*::mOrange^+^ cells and analyzed their expression by quantitative RT-PCR. We found that three genes putatively related to neural differentiation and function [*NVE25645* (neurexin-related), *NVE24524* (putative vesicular inhibitory amino acid transporter) and *NVE22970* (putative ionotropic glutamate receptor)] were downregulated in planula injected with *NvPrdm14d* shRNAs (Fig. 9). In contrast, the expression of *NvAtonal/neuroD* was upregulated (log2 fold change 1.30 and 0.59, respectively, for the two shRNAs, Fig. 9).

These results show that *NvPrdm14d* has both positive and negative roles in regulating the expression of genes enriched in *NvPrdm14d*::GFP^+^ neurons and that it is likely required for the acquisition of at least some neural features.

## Discussion

In this report, we find that all four *NvPrdm14* paralogs are expressed in the nervous system of *N. vectensis*. We show that the expression of *NvPrdm14d* defines a subpopulation of endodermal NPCs generating a subset of the endodermal/gastrodermal nervous system, including potential motoneurons in *Nematostella*. The overlap with *NvPOU4::mCherry* expression suggests that their terminal differentiation might be, in part, regulated by *NvPOU4*. Through the analysis of the *NvPrdm14d*::GFP^+^ transcriptome, we identified a panel of genes potentially involved in endodermal neurogenesis in *Nematostella*, either specifically in the *NvPrdm14d* lineage or more broadly. Finally, our functional analysis of *NvPrdm14d* revealed that this gene has positive and negative effects on neural gene expression in *Nematostella*.

### Endodermal origin of *NvPrdm14d*^+^ neurons and the heterogeneity of NPCs

The expression of *NvPrdm14d* is initially detectable in the pharynx before expression in scattered cells throughout the endoderm becomes visible (Fig. 1). One explanation for these dynamics would be a migration of neural progenitors or precursors from the pharynx into the body wall endoderm. Since the pharynx consists of an ectodermal and an endodermal part, the *NvPrdm14d*^+^ neurons in the body wall endoderm could potentially be of ectodermal origin. Two observations argue against this scenario. First, *NvPrdm14d* expression is predominantly found in the endodermal part of the pharynx (Fig. 2M) and second, the *NvPrdm14d*::GFP^+^ cells in the body wall endoderm do not express *NvFoxA*::mOrange2 (Fig. 7), which labels the ectodermal pharynx and cells derived from it. This indicates that most of the endodermal/gastrodermal *NvPrdm14d*^+^ neurons are indeed of endodermal origin, though we cannot exclude that some of them are derived from ectodermal pharyngeal cells.

Previous work has shown that ectodermal and endodermal neurogenesis in *Nematostella* are both regulated by transcription factors [*NvAth-like* and *NvSoxB(2)*] and signaling molecules (Notch) that act at the level of neural/secretory progenitor cells [N/SPCs^6,40,41^,]. The expression of *NvPrdm14d* in a subpopulation of *NvSoxB(2)*^+^ endodermal N/SPCs (Fig. 2) that gives rise only to neurons, shows that progenitor cell diversity, and not only terminal differentiation, is part of the endoderm-specific neurogenic program in *Nematostella*. We speculate that other populations of endodermal neurons derive from molecularly distinct progenitor cells and that the putative different types of neural progenitors can be considered as intermediate progenitor cells.

### The evolution of non-ectodermal neurogenesis

Non-ectodermal neurogenesis occurs in several taxa (Fig. 1A), but it is not well understood to what extend non-ectodermal neurogenesis differs from ectodermal neurogenesis within a given species and whether there are specific similarities in the regulation of non-ectodermal neurogenesis between different taxa. *SoxB* genes have been shown to be involved in ectodermal and non-ectodermal neurogenesis in sea urchin, *Nematostella* and mice, suggesting a general role in neurogenesis^6,7,9,11,22,40,86–88^. The identification of *NvPrdm14d* as a gene involved almost exclusively in endodermal neurogenesis in *Nematostella* provides an example for germ-layer specific differences in neurogenesis. While this observation is consistent with other cases of non-ectodermal neurogenesis, such as in the mesodermal pharyngeal neurons of *C. elegans* and the endodermal foregut neurons of the sea urchin^5,7^, we are not aware of conserved regulators that would be specific for non-ectodermal neurogenesis. The role of *Prdm14* in zebrafish motoneuron development^54^ shows that its function is not limited to non-ectodermal neurogenesis. However, the role of *Prdm14* in neurogenesis has been poorly described in general and it will be interesting to determine whether this gene is involved in non-ectodermal neurogenesis in other organisms. Differences in the regulation of ectodermal and non-ectodermal neurogenesis can be due to the embryonic origin of the neurons or to the neural cell types that are produced and broader investigations of non-ectodermal neurogenesis in cnidarians and bilaterians are, therefore, required to better understand the evolution of this process. The panel of candidate genes with a role potentially restricted to endodermal neurogenesis in *Nematostella* may serve as a resource for such studies.

### *NvPrdm14d*^+^ neurons as potential motoneurons

The localization of *NvPrdm14d*::GFP^+^ neurons in close vicinity of retractor muscles, their contact with putative post-synaptic sites of the retractor muscles and the expression of *NvIslet* (a direct target of Prdm14 in zebrafish motoneurons), indicate that *NvPrdm14d* ^+^ NPCs generate motoneurons in *Nematostella*. The transcriptome analysis of the *NvPrdm14d*::GFP^+^ cells identified additional transcription factors that might be involved in the development of these putative motoneurons, including some with roles in motoneuron development in other organisms [*e.g*. *Tbx20*^89^]. Functional analyses of the transcription factors identified here and in recent single cell sequencing studies^74,75^ will likely help identifying conserved and divergent regulators of motoneuron development. How the potential motoneurons are connected to other parts of the nervous system, remains unclear. The retractor muscle functions to retract the head and tentacles into the body column and accordingly the putative motoneurons might connect to the head region via neurites oriented along the oral-aboral axis within the mesenteries, rather than towards the body wall. We can, however, not exclude the possibility that we have missed connections towards the body wall in our sections or that they are established by a population of neurons that is not labeled by either the *NvPrdm14d*::GFP or the *NvElav1*::mOrange reporters.

In conclusion, we identified a population of endoderm-specific, *NvPrdm14d*-expressing neural progenitor cells whose progeny includes potential motoneurons. These observations provide new insights into the regulation of non-ectodermal neurogenesis and open new opportunities for a better understanding of nervous system evolution.

## Methods

### *Nematostella vectensis* culture

The *N. vectensis* culture is derived from CH2 males and CH6 females initially cultured by^90^. The culture is maintained at 18 °C in 1/3 filtered sea water [*Nematostella* medium (NM)], and spawned by a light and temperature shift (18–25 °C) for 12 h as previously described by ref. ^91^. Fertilized eggs are extracted from their jelly package by incubation in 3% cysteine in NM for 20 min with gentle shaking followed by four washes in NM. Embryos are then reared at 21 °C and fixed at 24 h post fertilization (hpf; early gastrula), 28 hpf (mid-gastrula), 30 hpf (late gastrula), 48 hpf (early planula), 60 hpf and 72 hpf (mid-planula), 96 hpf (late planula), 5 days post fertilization (dpf; early tentacle bud stage), 6 dpf (late tentacle bud stage), 10 dpf (primary polyp), or between 2 and 4 months post fertilization (mpf; growing polyps).

### Cloning of *NvPrdm14* paralogs, *NvAtonal/neuroD, NvAshD* and *NvPIT1*

The four *NvPrdm14* paralogs were identified by^72^ who established their phylogeny. Sequences were BLASTed to the *N. vectensis* NVE gene models (https://figshare.com/articles/Nematostella_vectensis_transcriptome_and_gene_models_v2_0/807696): *NvPrdm14a* (NVE22869, v1g104327), *NvPrdm14b* (NVE19092, v1g197426), *NvPrdm14c* (NVE9426, v1g61034), *NvPrdm14d* (NVE17327, v1g96522).

The mRNA material of pooled embryos at 24, 48 and 72 hpf were extracted using the RNAqueous^TM^ kit (AM1931). The SuperScript^TM^ III First-Strand Synthesis System (Invitrogen, 18080051) was used to generate cDNA from extracted mRNAs to perform PCR amplification with the primers listed in Supplementary Table 2. The cDNA of *NvPrdm14* paralogs were cloned into the pGEM®-T Easy vector system (Promega, A1360).

*NvAtonal/neuroD* (NVE18219, v1g220027), *NvAshD* (NVE2444, v1g210540) and *NvPIT1* (NVE1681, v1g112929) have been cloned following the same protocol.

Digoxigenin-labeled (DIG; Roche 11277073910) and fluorescein-labeled (Fluo; Roche 11685619910) riboprobes were synthetized from these clones with the MEGAscript^TM^ T7 or SP6 kit (Invitrogene, AMB1334/AMB1330).

### Colorimetric (ISH) and fluorescent (FISH) in situ hybridization

The following protocols are based on published methods^40,44^ with slight modifications.

Embryos were fixed in 3.7% formaldehyde/0.25% glutaraldehyde/NM for 1 min 30 s on ice, then in 3.7% formaldehyde/NM for 1 h at 4 °C. Embryos were washed in PBTw [Phosphate Buffered Saline (PBS) + 0.1% Tween20], in PBS, in H_2_O and stored at −20 °C in methanol.

For ISH, samples were rehydrated in PBTw, and then incubated in 20 µg/ml proteinase K for 5 min at room temperature (RT) followed by washes in 4 mg/ml glycine/PBTw. They were then washed in 1% triethanolamine in PBTw, followed by the addition of 0.25%, then 0.5% of acetic anhydride. Samples were next washed in PBTw, then refixed in 3.7% formaldehyde/PBTw, followed by washes in PBTw. Pre-hybridization was performed in hybridization buffer (50% formamide, 5× SSC, 1% SDS, 50 µg/ml heparin, 100 µg/ml salmon sperm DNA, 9.25 mM citric acid, 0.1X Tween20) at 60 °C overnight (ON). DIG-labeled riboprobes were incubated with the samples at a final concentration of 0.5 ng/µl at 60 °C for at least 60 h. Unbound probes were removed via a series of 60 °C washes of post-hybridization buffer (50% formamide, 5× SSC, 1% SDS, 0.1% Tween20, 9.25 mM citric acid) and 2× SSCT (SSC + 0,1% Tween20) solutions [75/25, 50/50, 25/75, 0/100 (v/v)], then 0.2× SSCT, 0.1X SSCT. This was followed by washes at RT in PBTw. Samples were then pre-blocked in blocking solution/PBTw [0.5% Block (Roche 11096176001), 50% PBTw, 50% maleic acid buffer (100 mM maleic acid, 150 mM NaCl)] for 5 min at RT, prior to be blocked 2 h at RT in 1% blocking solution/maleic acid buffer. Samples were then incubated with anti-DIG alkaline phosphatase (Roche, 1:4000)/blocking solution at 4 °C ON. Unbound antibodies were removed by 10 × 15 min washes of PBTx/BSA (PBS + 0.2% TritonX/0.1% bovine serum albumin); samples were then washed with staining buffer (100 mM Tris pH9.5, 100 mM NaCl, 50 mM MgCl_2_, 0.1% Tween20) before color was developed via addition of 1:100 NBT/BCIP solution (Roche, 11681451001) in staining solution. When the staining reaction was judged to be complete, samples were washed in staining buffer and H_2_O. Samples were cleared via ON incubation with 100% ethanol. Samples are rehydrated in H_2_O, then washed in PBTw and conserved in 87% glycerol at 4%. Samples were imaged on a Nikon Eclipse E800 compound microscope with a Nikon Digital Sight DSU3 camera. Images were cropped on Adobe Photoshop 2020 and some of them were made of assembled focal planes by focus stacking to show a better representation of the complete expression pattern. Figure plates were made on Adobe Illustrator 2020.

For single/double FISH, samples were incubated in 3% hydrogen peroxide/methanol to quench endogenous hydrogen peroxidase activity. Samples were then rehydrated in PBTw and the ISH protocol (see above) was followed from the proteinase K incubation step until the end of the SSCT washes at 60 °C. During hybridization, samples were incubated with either DIG or Fluo-labeled riboprobes at a final concentration of 0.5 ng/µl. After the SSCT washes at 60 °C, samples were washed in SSCT/PBTw solution at RT [75/25, 50/50, 25/75, 0/100 (v/v)], and then washed in TNTw (0.1 M Tris-HCl pH7.5, 0.15 M NaCl, 0.1% Tween20) and then blocked in TNT/block [0.5% blocking reagent (PerkinElmer FP1012)/TNT] for 1 h at RT before ON incubation with anti-DIG (1:100, Roche 11207733910) or anti-Fluo (1:250, Roche 11426346910) horseradish peroxidase. Unbound antibodies were removed by 10 × 15 min TNTx (0.1 M Tris-HCl pH7.5, 0.15 M NaCl, 0.2% TritonX) washes, and samples were then incubated in fluorophore tyramide amplification reagent (TSA^TM^ Plus kit, PerkinElmer, NEL748001KT). After the TSA reaction, samples were washed in TNTw and PBTw. For single FISH, samples were incubated with Hoechst 33342 [Thermo Fisher Scientific, 62249 (1:1000)] in PBTx and mounted in ProLong^TM^ Gold antifade reagent with DAPI (Thermo Fisher Scientific, P36935), while for double FISH, samples were then incubated with 0.1 M glycine pH2.0/0,1% Tween20 to inactivate the peroxidase activity of the first antibodies. Samples were then washed in PBTw and TNTw, before being incubated in TNTw/block for 1 h followed by an ON incubation with anti-DIG or anti-Fluo horseradish peroxidase (Roche). Post-antibody washes and the TSA reactions were repeated as for the first probe; samples were then washed in TNTx, in PBTx, incubated with Hoechst 33342 [Thermo Fisher Scientific, 62249 (1:1000)] in PBTx and mounted in ProLong^TM^ Gold antifade reagent with DAPI (Thermo Fisher Scientific, P36935). Samples were imaged on a Leica SP5 confocal microscope. Images were extracted from stacks with Fiji and adjusted for brightness/contrast and color balance, but any adjustments were applied to the whole image, not parts. Images were cropped on Adobe Photoshop 2020 and figures were made on Adobe Illustrator 2020.

### Cell proliferation assay

Embryos from desired stages were incubated with 100 µM EdU/DMSO in NM for 30 min or 2 h at RT and then fixed immediately as described above. Samples were then used for FISH. After the final washes in PBTx, EdU incorporation was visualized using the Click-iT^TM^ EdU imaging kit with Alexa Fluor^TM^ 647 (Thermo Fisher Scientific, C10424) following the manufacturer’s protocol. Samples were mounted and imaged the same way as for FISH. Images and figures were made the same way as well. The counting of *NvPrdm14d*^+^ and EdU^+^ cells was performed on serial 1 µm sections through the image stack of whole embryos.

### Generation of transgenic lines

The *NvPrdm14d::GFP* transgenic reporter line was generated by meganuclease-mediated transgenesis as described by^79^.

The genomic coordinates for the ca. 5 kb regulatory region are 226141-231086 on minus strand of scaffold 43 [http://genome.jgi.doe.gov/Nemve1/Nemve1.home.html, accessed in September 2020^92^]. This region was cloned in front of a codon optimized *gfp* gene with the addition of a membrane tethering CAAX domain to help visualizing the boundaries and morphology of cells expressing the reporter protein. This reporter cassette was flanked by inverted I-Sce1 sites and cloned into the pUC57 vector backbone (GenScript). Wild-type fertilized eggs were injected with a mix containing: plasmid DNA (20 ng/µl), I-Sce1 (1 U/µl, NEB R0694), Dextran Alexa Fluor^TM^ 568 (100 ng/µl) and CutSmart buffer (1×). The mix was incubated at 37 °C for at least 15 min, then injection was performed with a FemtoJet® 4i microinjector (Eppendorf). The genomic coordinates for the *FoxA* promoter are 459458–465367 on the minus strand of scaffold 58. The promoter fragment was cloned with AscI and PacI into the transgenesis vector described in ref. ^78^ and the line was generated as described above. For the *NvMyHC1::homer-mCherry* line, the open reading frame of a *Nematostella homer* gene (GenBank XM_032362161) was cloned with AscI/AscI into the *NvMyHC1::mCherry* plasmid^78^. In the resulting fusion protein, NvHomer is tagged at the C-terminus with mCherry. Constructs and/or transgenic lines are available from the authors upon request.

### Immunofluorescence (IF)

For immunostainings, embryos were fixed in 3.7% formaldehyde/0.25% glutaraldehyde/NM for 1 min 30 s on ice, then in 3.7% formaldehyde/NM for 1 h at 4 °C. Embryos were washed several times in PBTw and in PBTx, and stored at 4 °C in PBTx for short periods of time (no more than a week). Embryos older than 72 hpf and growing polyps were anesthetized with MgCl_2_ before fixation to prevent muscular contraction. Growing polyps were quickly killed by 30 µl/ml 3.7% fomaldehyde/NM before fixation and fixed in 3.7% formaldehyde/NM ON (instead of 1 h) at 4 °C. Prior to performing IF, the extremity of growing polyp physa was cut to allow a proper staining of mesenteries.

For IF, samples were washed several times in PBTx during 2 h at RT, blocked in blocking solution [3% BSA/5% Normal Goat Serum (NGS)/PBTx] for 1 h at RT and incubated with primary antibodies in blocking solution ON at 4 °C. Primary antibodies used are: anti-GFP [mouse abcam ab1218 (1:200) or rabbit abcam ab290 (1:200)], and anti-DsRed [rabbit Clontech 632496 (1:100)] or anti-mCherry [mouse Clontech 632543 (1:100)]. Samples were then washed extensively in PBTx (5 washes during at least 2 h 30 min) at RT, blocked in blocking solution for 1 h at RT and incubated with secondary antibodies in blocking solution ON at 4 °C. Secondary antibodies used are: anti-mouse Alexa 488 [goat, Life Technologies A11001 (1:200)], anti-mouse Alexa 568 [goat, Life Technologies A11004 (1:200)], anti-rabbit Alexa 488 [goat, Life Technologies A11008 (1:200)], anti-rabbit Alexa 568 [goat, Life Technologies A11011 (1:200)]. If phalloidin staining was performed, Alexa Fluor^TM^ 633 phalloidin [Thermo Fisher Scientific, A22284, (1:50)] was incubated at the same time. Samples were then washed extensively in PBTx (5 washes during at least 2 h 30 min) at RT, incubated with Hoechst 33342 [Thermo Fisher Scientific, 62249 (1:1000)] in PBTx, washed extensively 5 × 10 min in PBTx, and mounted in ProLong^TM^ Gold antifade reagent with DAPI (Thermo Fisher Scientific, P36935). Samples were imaged on a Leica SP5 confocal microscope. Images were extracted from stacks with Fiji and adjusted for brightness/contrast and color balance, but any adjustments were applied to the whole image, not parts. Images were cropped on Adobe Photoshop 2020 and figures were made on Adobe Illustrator 2020.

### Vibratome sectioning

After immunostaining (see above), whole growing polyps were embedded in gelatin/albumin medium [0.4% gelatin type A (Sigma G1890), 27% albumin (Sigma, A3912), 3.7% formaldehyde, in PBS] at RT. Sectioning was performed at RT in PBTx on a Leica VT1000S vibratome. Sections had a thickness of either 20 µm, 50 µm or 100 µm. Sections were stored in PBTx at 4 °C and mounted in ProLong^TM^ Gold antifade reagent with DAPI (Thermo Fisher Scientific, P36935).

Sections were imaged on a Leica SP5 or SP6 confocal microscope. Images were extracted from stacks with Fiji and adjusted for brightness/contrast and color balance, but any adjustments were applied to the whole image, not parts. Images were cropped on Adobe Photoshop 2020 and figures were made on Adobe Illustrator 2020.

### Fluorescence-activated cell sorting (FACS)

Cells were sorted by a fluorescence-activated cell sorter as previously described by ref. ^93^, the gating strategy is depicted in Supplementary Fig. 5.

About a thousand primary polyps positive for *NvPrdm14d*::GFP at 13dpf were dissociated in 0.25% Trypsin (Gibco, 27250018) in Ca- Mg-free NM (CMFNM; 154 mM NaCl, 3.6 mM KCl, 2.4 mM Na_2_SO_4_, 0.7 mM NaHCO_3_) supplemented with 6.6 mM EDTA (pH7.6–7.8) at 37 °C for 30 min. Cells were centrifuged at 800 × *g* for 10 min, resuspended in ice cold 0.5% BSA in CMFNM (pH7.6–7.8), filtered through a 40 µm filter and stained with Hoechst 33342 (Thermo Fisher Scientific, 62249) at a concentration of 60 µg/ml at RT for 30 min. Samples were then diluted 1:1 with ice cold 0.5% BSA/CMFNM and stained with 60 µg /ml 7-AAD (BD, 559925) for more than 20 min on ice. Cell sorting was performed using BD FACSDiva v8.0 on a BD FACSAria II with a 100 µm nozzle.

### RNA extraction and sequencing

The RNAseq of *NvPrdm14d*::GFP^+^ cells was performed as previously described in^93^. After FACS, GFP^+^ and GFP^-^ cells were collected in 0.5% BSA in CMFNM (pH7.6–7.8) and centrifuged at 800 × *g* and 4 °C for 10 min. Most of the liquid was removed and cells were resuspended in TRIzol^TM^ LS reagent (3:1 reagent to sample ratio, Invitrogen 10296028) in 3 independent sorts. Samples were vortexed extensively and incubated at RT for 5 min before being flash frozen and stored at −80 °C. The samples were processed using Direct-zol^TM^ RNA MicroPrep columns (Zymo Research, R2060) following the manufacturer’s protocol, including on column DNAse digestion. The RNA quality was assessed using an RNA 6000 Pico kit (Agilent, 5067–1513) on a Bioanalyzer (Agilent G2939A).

Libraries were generated using the NEBNext® Ultra™ II Directional RNA Library Prep kit for Illumina® (NEB #7760L) with 2.5 ng RNA input (1/300 adaptor dilution, 20 PCR cycles). Libraries were sequenced using a 75 bp single end sequencing on a NextSeq® 500 machine (Illumina).

### Bioinformatic analysis of the *NvPrdm14d* transcriptome

The quality control of raw RNAseq-reads was initially assessed with FastQC software v.0.11.8 (http://www.bioinformatics.babraham.ac.uk/projects/fastqc/). Raw reads were then filtered with fastp v.0.20.0^94^ using default settings and mapped with STAR aligner v.2.7.3a^95^ with default settings to the *N. vectensis* genome (http://genome.jgi.doe.gov/Nemve1/Nemve1.home.html)^92^ using NVE gene models (https://figshare.com/articles/Nematostella_vectensis_transcriptome_and_gene_models_v2_0/807696). Downstream analysis were performed using R v.4.0.2 (https://www.r-project.org/) and Bioconductor packages (https://www.bioconductor.org/). Aligned reads were counted with “summarizedOverlaps” function from the R package “GenomicAlignments” v.1.24.0^96^ and only genes with at least 10 counts in at least 3 biological replicates for one condition were kept. Differential expression analysis was performed using the package “DESeq2” v.1.28.1^97^. Overlaps between up- and/or downregulated genes across different conditions were assessed using the package “GeneOverlaps” v.1.23.0 (http://shenlab-sinai.github.io/shenlab-sinai/). GO terms enrichment was assessed using the package “ClusterProfiler” v.3.16.0^98^.

### shRNA-mediated knockdown of *NvPrdm14d*

shRNAs were designed and synthesized based on published protocols^85,99^ with slight modifications, the primers are listed in Supplementary Table 3.

Each primer pair was mixed to a final concentration of 20 µM in a total volume of 20 µl, heated to 98 °C for 2 min before slow cooling to room temperature. These solutions (5.5 µl each) were then used as template for a 20 µl reaction with the AmpliScribe^TM^ T7 Transcription Kit (Lucigen, AS3107). The reaction product was treated with DNAseI and shRNAs were purified using the RNA Clean and Concentrator^TM^ Kit (Zymo Reasearch, R1012).

Wild-type fertilized eggs were injected with a mix containing: shRNA (900 ng/µl) and Dextran Alexa Fluor^TM^ 488 (100 ng/µl). The mix was injected with a FemtoJet® 4i microinjector (Eppendorf).

### RNA isolation and qPCR

RNAs from *NvPrdm14d* knockdown were isolated based on a published protocol^76^ with slight modifications.

Animals injected with either shRNA #1 or #2 were collected at 48 hpf and lysed in 500 µl of TRIzol^TM^ reagent (Invitrogen, 15596026) by vortexing extensively, and incubated at room temperature for 5 min. Chloroform (135 µl) was added and samples were vigorously mixed. The aqueous phase was isolated using MaXtract® High Density tubes (QIAGEN, 129046) according to the manufacturer’s protocol. One volume of 100% ethanol was added to the aqueous phase. This RNA-containing solution was processed using the RNeasy® Mini Kit (QIAGEN, 74104) according to the manufacturer’s instructions, including on column DNAseI digestion using the RNAse-Free DNAse Set (QIAGEN 79254). The SuperScript^TM^ III First-Strand Synthesis System (Invitrogen, 18080051) was used to generate complementary DNA, and it was primed with random hexamers (Custom oligos, Sigma-Aldrich).

qPCRs were performed using the QuantiTect^TM^ SYBR® Green PCR Master mix (QIAGEN, 204143) and ran on a Bio-Rad CFX96 system. Primers are listed in Supplementary Table 4.

### Statistics and reproducibility

Samples sizes were determined empirically; no data were excluded. The investigators were not blinded to allocation during experiments and outcome assessment; the experiments were analyzed by two independent researchers. For all in situ hybridizations, immunohistochemistry experiments and EdU assays, the images shown in the figures are representative for > 60% of the specimens for each replicate. The number of replicates are: 3–7 (Fig. 1), 2–3 (Fig. 2), 2–17 (Fig. 3), 2–4 (Fig. 4), 2–4 (Fig. 5), 3–5 (Fig. 6), 2 (Fig. 7), 2 (Fig. 8), 2–3 (Supplementary Fig. 2), 2 (Supplementary Fig. 3).

For RNA seq analysis (Fig. 8C), differentially expressed genes were identified using DESeq2 package, which employs negative binomial generalized linear models and provides Wald test *p*-values adjusted for multiple testing using the Benjamini Hochberg correction. *P*-adjusted values for differentially expressed genes are provided in Supplementary Data 1. Enriched GO terms (Supplementary Data 3) were obtained using the “enricher” function of the clusterProfiler R package and *p-*values were adjusted for multiple comparisons using the Benjamini Hochberg method. Statistical tests and support for other analyses are provided in the figure legends.

### Reporting summary

Further information on research design is available in the Nature Portfolio Reporting Summary linked to this article.

### Supplementary information

Supplementary Information

Description of Additional Supplementary Files

Supplementary Data 1

Supplementary Data 2

Supplementary Data 3

Reporting Summary

### Source data

Source Data

## Data Availability

The RNA-sequencing raw data for *NvPrdm14d*::GFP cells are deposited as NCBI BioProject with accession number PRJNA962287. Lists of genes derived from the analyses presented in Fig. 8 are available as Supplementary Data 1–3. Source data are provided with this paper.
