# Supplementary material for: NvPrdm14d-expressing neural progenitor cells contribute to non-ectodermal neurogenesis in Nematostella vectensis
Source: Nat Commun. 2023 Aug 10;14:4854. doi: 10.1038/s41467-023-39789-4 (PMC10415408; doi:10.1038/s41467-023-39789-4)
Supplement: Supplementary file 1 — Supplementary Information [file 41467_2023_39789_MOESM1_ESM.pdf]

SUPPLEMTARY INFORMATION

"*NvPrdm14d*-expressing neural progenitor cells contribute to non-ectodermal neurogenesis in *Nematostella vectensis*"

Content:

Supplementary Figure 1: Expression of *NvPrdm14d* and *NvElav1* in published single cell sequencing data.

Supplementary Figure 2: *NvPrdm14d*::GFP$^+$ neurons are closely associated with retractor muscles but not connected to the body wall nerve net through *NvPrdm14d*::GFP$^+$ or *NvElav1*::mOrange$^+$ neuron.

Supplementary Figure 3: A transgenic reporter line for *NvMyHC1*::homer-mCherry highlights putative post-synaptic sites in the retractor muscles.

Supplementary Figure 4: Expression pattern of *NvAtonal/neuroD* by colorimetric *in situ* hybridization.

Supplementary Figure 5: Gating strategy for sorting cells from *NvPrdm14d::GFP* polyps.

Supplementary Table 1: Detection of the four *NvPrdm14* genes in available transcriptome data

Supplementary Table 2: List of primers used for cloning genes.

Supplementary Table 3: List of primers for generating shRNAs.

Supplementary Table 4: List of primers used for qPCR.

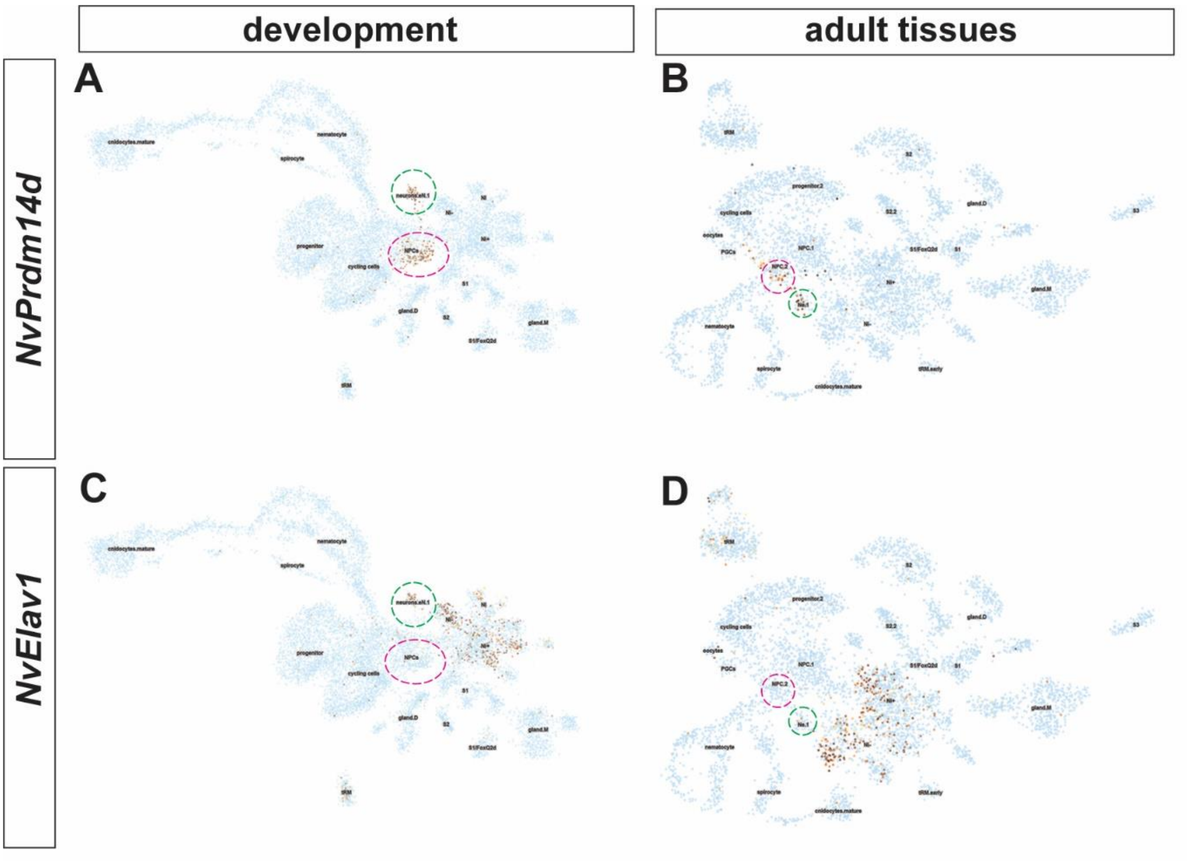

Supplementary Figure 1: Expression of *NvPrdm14d* and *NvElav1* in published single cell sequencing data. UMAP plots of cells of the neuroglandular lineage at developmental stages (A, C, including stages from planula to primary polyp), and adult tissues (B, D). Cells expressing *NvPrdm14d* (A, B) or *NvElav1* (C, D) are labelled in yellow and brown. Cell populations annotated as neural progenitor cells are highlighted by a dashed line in magenta, cells annotated as "endodermal neurons" are highlighted by a dashed line in green. At developmental stages, *NvPrdm14d* and *NvElav1* are expressed in the "endodermal neurons", but only *NvPrdm14d* is expressed in neural progenitors. In adult tissue, only very few *NvPrdm14d*-expressing "endodermal neurons" express *NvElav1*. Data from ref. 75, analyzed at https://cells.ucsc.edu/?ds=sea-anemone-atlas+neurosec+dev

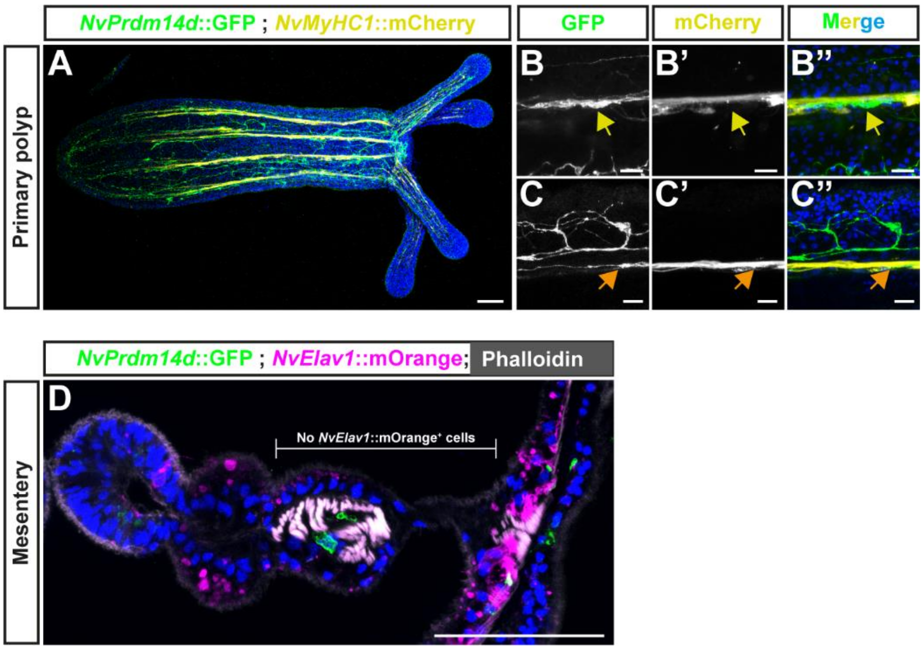

Supplementary Figure 2: *NvPrdm14d*::GFP⁺ neurons are closely associated with retractor muscles but not connected to the body wall nerve net through *NvPrdm14d*::GFP⁺ nor *NvElav1*::mOrange⁺ neurons. (A-C) Confocal images of immunofluorescence staining for *NvPrdm14d::*GFP and *NvMyCH1*::mCherry, respectively shown in green and yellow. At this stage, neurites expressing *NvPrdm14d*::GFP run along the retractor muscles. Arrows in (B-C) indicate *NvPrdm14d*::GFP⁺ neurites associated to retractor muscles. (D) Confocal image of immunofluorescence staining for *NvPrdm14d:*:GFP, *NvElav1*::mOrange and Phalloidin in mesenteries. GFP is shown in green, mOrange in magenta and muscles in white. No *NvElav1*::mOrange⁺ neurons is present in the mesenteries between the body wall and the retractor muscles.

Samples are counterstained for DNA in blue. The oral pole of the primary polyp is oriented to the right. Scale bars: 50 µm in (A & D) and 10 µm in (B-C).

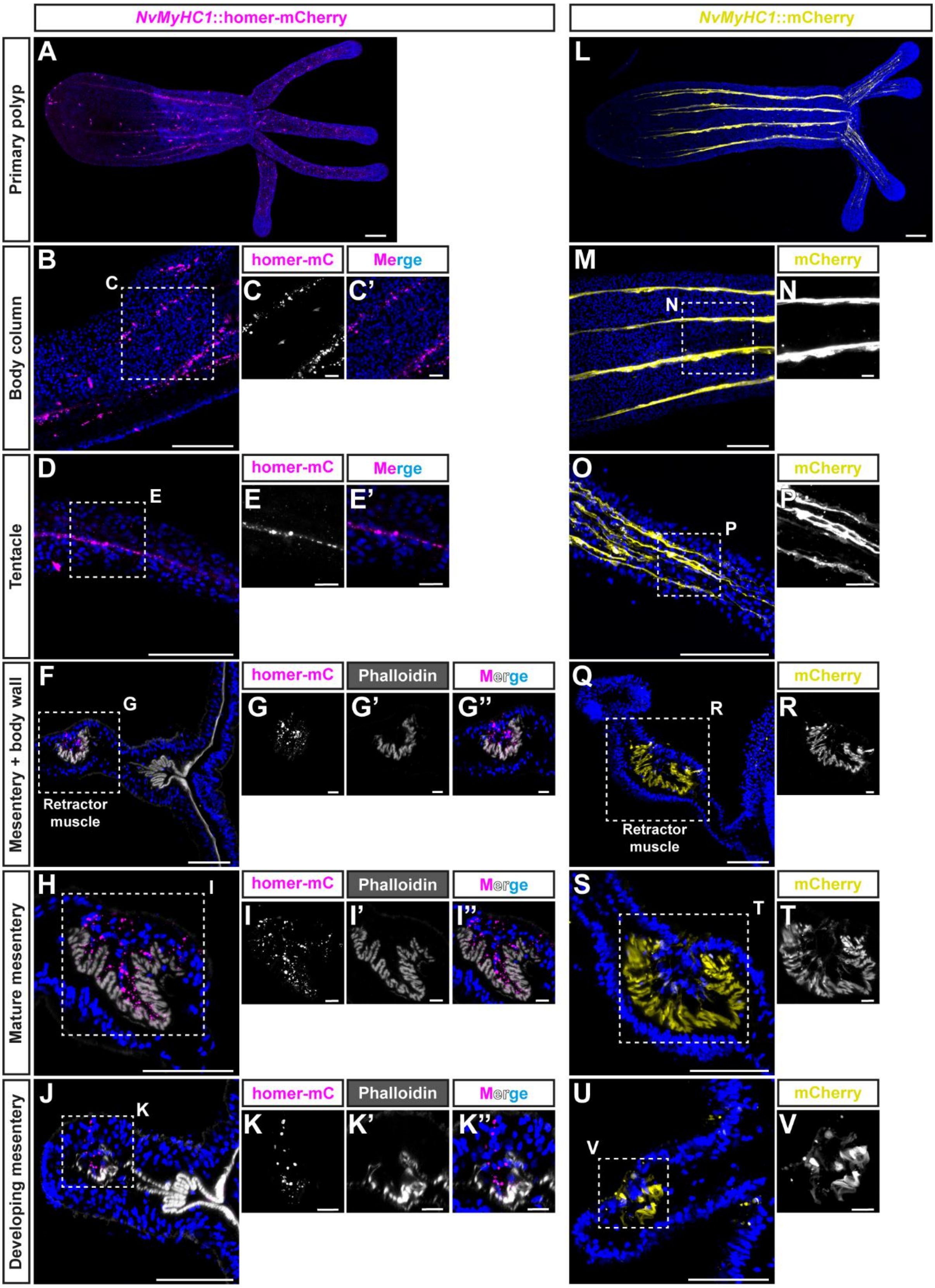

Supplementary Figure 3: A transgenic reporter line for *NvMyHC1*::homer-mCherry highlights putative post-synaptic sites in the retractor muscles. (A-V) Confocal images of immunofluorescence staining for *NvMyHC1*::homer-mCherry (magenta) and *NvMyHC1*::mCherry (yellow) in primary polyps (A & L), body column (B-C & M-N), tentacles (D-E & O-P), and different types of mesenteries (F-K & Q-

V). The Homer-mCherry fusion protein is expressed in puncta along the longitudinal tracts of the primary polyp (A), as do retractor muscles shown via the expression of the mCherry protein (L). This linear arrangement of Homer-mCherry$^+$ puncta is found in both the body column and the tentacles (B-E), a location where mCherry$^+$ retractor muscles are found (M-P). Cross-sections reveal that Homer-mCherry$^+$ puncta are exclusively found in mesenteries (F), more precisely, associated with retractor muscles (G-K). As previously shown by (ref. 78), retractor muscles are the only structure labelled by the mCherry protein and they do not exhibit a pattern composed of mCherry$^+$ puncta, whatever the developmental stage of mesenteries (Q-V). Therefore, the *NvMyHC1*::homer-mCherry reporter line is seemingly labelling post-synaptic sites specifically in the retractor muscles.

Samples are counterstained for DNA in blue. In (F-K), muscles are stained in white by phalloidin. The oral pole of primary polyps is oriented to the right. Scale bars: 50 µm in (A, B, D, F, H, J, L, M, O, Q, S & U), 10 µm in (C, E, G, I, K, N, P, R, T & V).

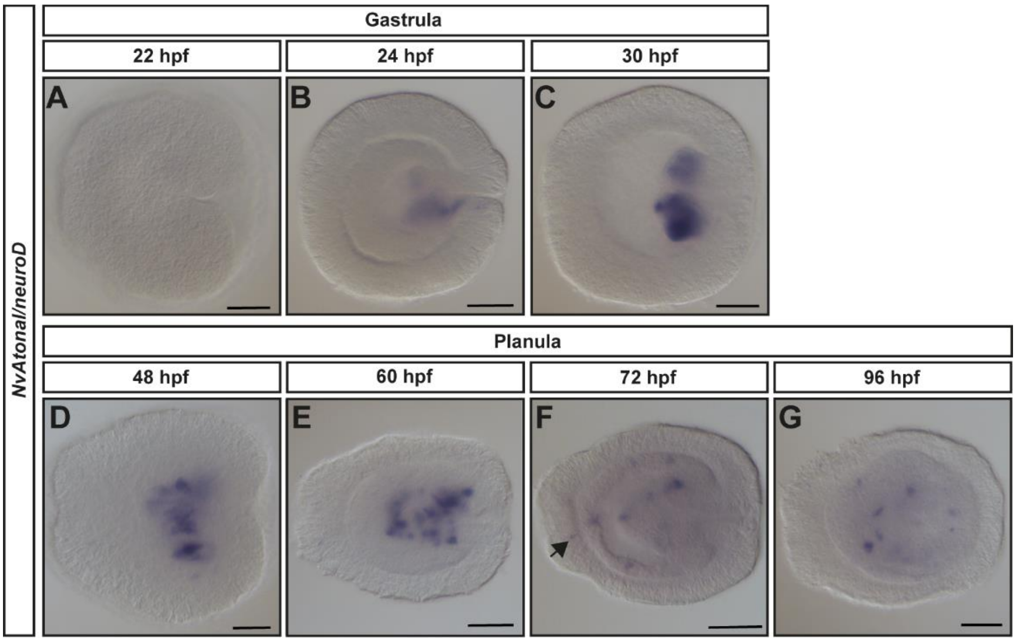

Supplementary Figure 4: Expression pattern of *NvAtonal/neuroD* by colorimetric in situ hybridization. (A) In the early gastrula, *NvAtonal/neuroD* is not expressed. (B) In mid-gastrula, *NvAtonal/neuroD* starts to be expressed in the pharynx. Strong expression in the pharynx persists until mid-planula (E). In mid-planula (F), *NvAtonal/neuroD* starts to be expressed in scattered endodermal cells, while the strong expression in the pharynx disappears. Few ectodermal cells express *NvAtonal/neuroD* (arrow in F). From this stage, the expression remains in scattered cells within the pharynx and the endoderm (F-G). This expression pattern resembles the one of *NvPrdm14d* but in fewer cells, suggesting a role for *NvAtonal/neuroD* in the development of a subset of endodermal *NvPrdm14d*$^{+}$ cells/neurons. In all pictures, the oral pole is oriented to the right. Scale bars: 50 µm

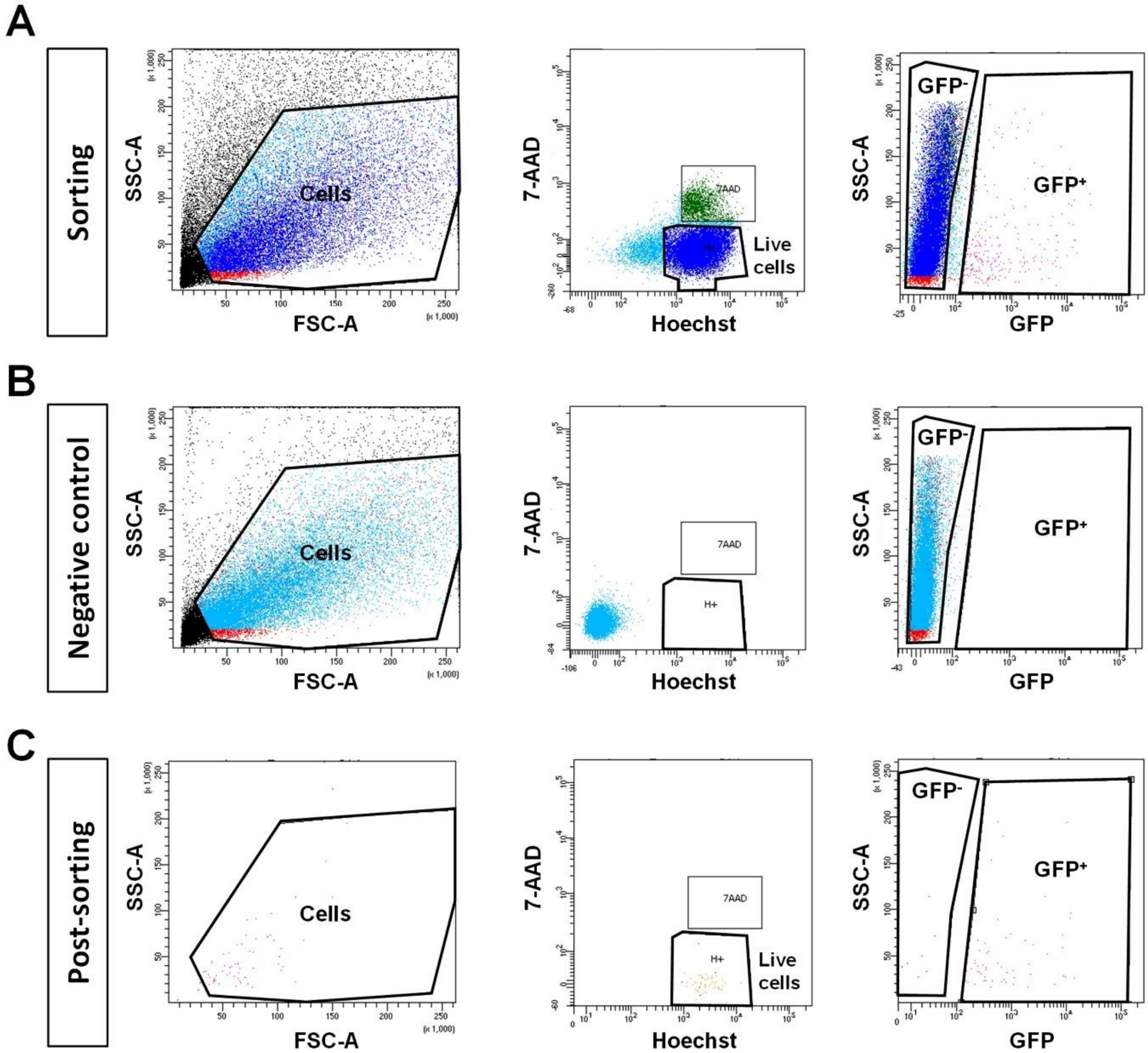

Supplementary Figure 5: Gating strategy for sorting cells from *NvPrdm14d::GFP* polyps. (A) FlowJo plots showing the gating strategy used to select cells during the Fluorescence Activated Cell Sorting (FACS) of *NvPrdm14d*::GFP^{+} vs. *NvPrdm14d*::GFP^{-} cells. (B) FlowJo plots showing that no cell is sorted as GFP^{+} with our gating strategy when unstained cells from wild-type polyps are used. (C) FlowJo plots showing the flow cytometry analysis of sorted GFP^{+} cells. After sorting, cells are confirmed to be alive and GFP^{+}.

Supplementary Table 1: Detection of NvPrdm14 genes in available transcriptomic data. The green color indicates where each gene is detected. For the transcriptome of *NvPOU4* mutants, it is indicated whether the gene is up- or downregulated. The ID of clusters from the single-cell atlas is indicated when genes are detected. *NvElav1*::mOrange$^+$ and *NvPOU4*$^{-/-}$ transcriptomes from (ref. 44), NvNCol3$^+$ transcriptome from (ref 73), *NvPrdm14d*::GFP$^+$ transcriptome from this paper, and single-cell clusters from (ref. 74).

***NvPrdm14* genes**	**Transcriptomes**				**Single-cell clusters**			
	NvElav1$^+$	***NvNCol3***$^+$	***NvPrdm14d***$^+$	***NvPOU4***$^{-/-}$	**Neuronal**	**Cnidocyte**	**Muscle Gastrodermis**	**Gland Secretory**
NvPrdm14a (NVE22869, v1g104327)				**DOWN**				
NvPrdm14b (NVE19092, v1g197426)								
NvPrdm14c (NVE9426, v1g61034)					**C34**			
NvPrdm14d (NVE17327, v1g96522)					**C35 & C36**			

Supplementary Table 2: List of primers used for cloning genes.

NvPrdm14a	Primer forward: ATATGCCGTCCTTGGAACAG
	Primer reverse: GGCCTATTCCTGCATTTTCA
NvPrdm14b	Primer forward: AGGCTGCTGGGACAGATAAA
	Primer reverse: ACTTGTATGGCCTCGTACCG
NvPrdm14c	Primer forward: GGCCAATCACACAATAGAAGC
	Primer reverse: CCTTGCACACAAACCAACTG
NvPrdm14d	Primer forward: CCGCAAAATGGCAGAGCTGC
	Primer reverse: GTCATTTGAGATCAGATCCTC
NvAtonal/neuroD	Primer forward: GAGACCTTTGCGAACCACAGCC
	Primer reverse: GTCATTCAGTGTTTCGGTGACGATC
NvAshD	Primer forward: CAATCATGAGCGAAGTGATAGAG
	Primer reverse: GATAACTCGAGTTAAGCGGAG
NvPIT1	Primer forward: CAGCATTATTGTTTGGCCGG
	Primer reverse: GCATCTGATTCCTTACTGCGG

Supplementary Table 3: List of primers used to synthetize the shRNAs targeting *NvPrdm14d*. The sequence of the shRNA is marked in bold. Regular font in primers indicates the T7 promoter sequence and the italic font marks the sequence of the loop found in the final shRNA.

shRNA #1	Sequence: **GACCGCCTGAGAATTCATA**
	Primer forward: TAATACGACTCACTATA**GACCGCCTGAGAATTCATA***TTCAAGAGA***TATGAATTCTCAGGCGGTC***TT*
	Primer reverse: *AA***GACCGCCTGAGAATTCATA***TCTCTTGAA***TATGAATTCTCAGGCGGTC**TATAGTGAGTCGTATTA
shRNA #2	Sequence: **GCCTCAACAAGCATCTAAG**
	Primer forward: TAATACGACTCACTATA**GCCTCAACAAGCATCTAAG***TTCAAGAGA***CTTAGATGCTTGTTGAGGC***TT*
	Primer reverse: *AA***GCCTCAACAAGCATCTAAG***TCTCTTGAA***CTTAGATGCTTGTTGAGGC**TATAGTGAGTCGTATTA
control shRNA	Sequence: GTGGAGTTGGACGGAGATGT
	Primer forward: TAATACGACTCACTATA**GTGGAGTTGTAAGGAGCTGT***TTCAAGAGA*
	Primer reverse: *AA***GTGGAGTTGGACGGAGATGT**TCTCTTGAAACAGCTCCTTACAA

Supplementary Table 4: List of primers used for qPCR.

NvPrdm14d	Primer forward: TGGGAGATCTTTCAAGATGGCGAA
	Primer reverse: TCATCGTTATCGGGATTCCCATGT
NvAtonal/neuroD	Primer forward: AAACGTACGAAGCTAACAGTTCC
	Primer reverse: AAATAGTCTGGAATTACGCCTCG
NVE25645 (Neurexin-2 related)	Primer forward: TTGTCTTGAGTGCTGGCGATGG
	Primer reverse: TATCAGATTGGAAGAGGTACCAGGG
NVE24524 (Vesicular inhibitory amino acid transporter)	Primer forward: TGAGCAAGGGTGGCTACATGG
	Primer reverse: GCTTTCCGTCAGCGTTCATGG
NVE22970 (Glutamate receptor)	Primer forward: GGAACTCCGAGTGGACAGATG
	Primer reverse: GCAGTTCTAGACTCATTGGCTGG
