# Supplementary material for: NvPrdm14d-expressing neural progenitor cells contribute to non-ectodermal neurogenesis in Nematostella vectensis
Source: Nat Commun. 2023 Aug 10;14:4854. doi: 10.1038/s41467-023-39789-4 (PMC10415408; doi:10.1038/s41467-023-39789-4)
Supplement: Supplementary file 2 — Description of Additional Supplementary Files [file 41467_2023_39789_MOESM2_ESM.pdf]

Description of Additional Supplementary Files

File Name: Supplementary Data 1
Description: List of genes differentially expressed in *NvPrdm14d*::GFP$^+$ cells. Upregulated genes are labelled in red and it is specified when they are part of the stringent list. Downregulated genes are labelled in blue. *NvPrdm14d* is highlighted in green.

File Name: Supplementary Data 2
Description: List of genes defining the *NvPrdm14d*$^+$ neuronal metacells. Among the genes defining the neuronal metacell 35, 73.47% are found in the *NvPrdm14d*::GFP$^+$ transcriptome, while 77.16% of the genes defining the neuronal metacell 36 are found in the *NvPrdm14d*::GFP$^+$ transcriptome. *NvPrdm14d* is highlighted in green. Extracted from reference 74.

File Name: Supplementary Data 3
Description: List of upregulated GO terms in the *NvPrdm14d*::GFP$^+$ cells. Stringent terms are mostly related to receptors, channels, and transmembrane transport. After exclusion of genes upregulated in both *NvPrdm14d*::GFP$^+$ and *NvElav1*::mOrange$^+$ cells, enriched GO terms are mostly related to chromosome organization and cell cycle regulation. The six GO terms related to neuromuscular functions are highlighted in light blue.
