# Supplementary material for: NvPrdm14d-expressing neural progenitor cells contribute to non-ectodermal neurogenesis in Nematostella vectensis
Source: Nat Commun. 2023 Aug 10;14:4854. doi: 10.1038/s41467-023-39789-4 (PMC10415408; doi:10.1038/s41467-023-39789-4)
Supplement: Supplementary file 6 — Reporting Summary [file 41467_2023_39789_MOESM6_ESM.pdf]

Corresponding author(s): Fabian Rentzsch

Last updated by author(s): Jun 16, 2023

Reporting Summary

Nature Portfolio wishes to improve the reproducibility of the work that we publish. This form provides structure for consistency and transparency in reporting. For further information on Nature Portfolio policies, see our Editorial Policies and the Editorial Policy Checklist.

Statistics

For all statistical analyses, confirm that the following items are present in the figure legend, table legend, main text, or Methods section.

n/a	Confirmed	
☐	☒	The exact sample size (*n*) for each experimental group/condition, given as a discrete number and unit of measurement
☐	☒	A statement on whether measurements were taken from distinct samples or whether the same sample was measured repeatedly
☐	☒	The statistical test(s) used AND whether they are one- or two-sided<br>*Only common tests should be described solely by name; describe more complex techniques in the Methods section.*
☒	☐	A description of all covariates tested
☐	☒	A description of any assumptions or corrections, such as tests of normality and adjustment for multiple comparisons
☐	☒	A full description of the statistical parameters including central tendency (e.g. means) or other basic estimates (e.g. regression coefficient) AND variation (e.g. standard deviation) or associated estimates of uncertainty (e.g. confidence intervals)
☐	☒	For null hypothesis testing, the test statistic (e.g. *F*, *t*, *r*) with confidence intervals, effect sizes, degrees of freedom and *P* value noted<br>*Give P values as exact values whenever suitable.*
☒	☐	For Bayesian analysis, information on the choice of priors and Markov chain Monte Carlo settings
☒	☐	For hierarchical and complex designs, identification of the appropriate level for tests and full reporting of outcomes
☒	☐	Estimates of effect sizes (e.g. Cohen's *d*, Pearson's *r*), indicating how they were calculated

Our web collection on statistics for biologists contains articles on many of the points above.

Software and code

Policy information about availability of computer code

Data collection	BD FACSDIVA on the BD FACSARIA II v9.1.2, and Bio-Rad CFX96
Data analysis	FastQC software v.0.11.8, fastp v.0.20.0 , STAR aligner v.2.7.3a , R v.4.0.2, Bioconductor packages (GenomicAlignments v.1.24.0 , DESeq2 v.1.28.1 , GeneOverlaps v.1.23.0 , ClusterProfiler v.3.16.0)

For manuscripts utilizing custom algorithms or software that are central to the research but not yet described in published literature, software must be made available to editors and reviewers. We strongly encourage code deposition in a community repository (e.g. GitHub). See the Nature Portfolio guidelines for submitting code & software for further information.

Data

Policy information about availability of data

All manuscripts must include a data availability statement. This statement should provide the following information, where applicable:

- Accession codes, unique identifiers, or web links for publicly available datasets
- A description of any restrictions on data availability
- For clinical datasets or third party data, please ensure that the statement adheres to our policy

The RNA-sequencing raw data for NvPrdm14d::GFP cells are deposited as NCBI BioProject with accession number PRJNA962287. Lists of genes derived from the analyses presented in Figure 8 are available as Supplementary Datasets 1-3.
The different animal silhouttes for the phylogeny in figure 1A were found on Phylopic (https://phylopic.org/) and we have only used those that are dedicated to the

public domain.
The Prdm14d paralogs, NvAtonal/neuroD, NvAshD and NvPIT1 sequences used in the manuscript were retrieved from the N. vectensis NVE gene models (https://figshare.com/articles/Nematostella_vectensis_transcriptome_and_gene_models_v2_0/807696). All accession numbers are listed in the material and methods section.
The genomic coordinates for the ca. 5kb regulatory region of NvPrdm14d used for generating the transgenic reporter line are 226141-231086 on minus strand of scaffold 43 accessed in September 2020 (http://genome.jgi.doe.gov/Nemve1/Nemve1.home.html).
The NvElav1::mOrange transcriptome data is previously published with accession number E-MTAB-8794.
The NvNCol3::mOrange transcriptome data is previously published with accession number E-MTAB-9556.
The NvPOU4 mutant transcriptome data is previously published with accession number E-MTAB-8658.
No new code was generated for this study.

Human research participants

Policy information about studies involving human research participants and Sex and Gender in Research.

Reporting on sex and gender	No human research participants were used in this study.
Population characteristics	No human research participants were used in this study.
Recruitment	No human research participants were used in this study.
Ethics oversight	No human research participants were used in this study.

Note that full information on the approval of the study protocol must also be provided in the manuscript.

Field-specific reporting

Please select the one below that is the best fit for your research. If you are not sure, read the appropriate sections before making your selection.

☒ Life sciences ☐ Behavioural & social sciences ☐ Ecological, evolutionary & environmental sciences

For a reference copy of the document with all sections, see nature.com/documents/nr-reporting-summary-flat.pdf

Life sciences study design

All studies must disclose on these points even when the disclosure is negative.

Sample size	No specific sample size calculation was performed. The number of embryos taken per biological replicate used for subsequent RNASeq was always approximately 1000. This number of embryos yields sufficient amount of RNA for Illumina library preparation after FACS.<br>Sample size for in situ hybridization and immunohistochemistry experiments (ca 20-100 specimens per sample) is empirically determined to allow high quality and reproducibility.
Data exclusions	No data was excluded.
Replication	The number of biological replicates for RNASeq experiments was 3. All other experiments were replicated at least twice. Only biological replicates were used. All attempts at replication were successful.
Randomization	The groups were in all cases determined by the genotype of the animals.
Blinding	No blinding was performed. The outcomes of the experiments were evaluated by at least two independent researchers.

Reporting for specific materials, systems and methods

We require information from authors about some types of materials, experimental systems and methods used in many studies. Here, indicate whether each material, system or method listed is relevant to your study. If you are not sure if a list item applies to your research, read the appropriate section before selecting a response.

Materials & experimental systems

n/a	Involved in the study
☐	☒ Antibodies
☒	☐ Eukaryotic cell lines
☒	☐ Palaeontology and archaeology
☐	☒ Animals and other organisms
☒	☐ Clinical data
☒	☐ Dual use research of concern

Methods

n/a	Involved in the study
☒	☐ ChIP-seq
☐	☒ Flow cytometry
☒	☐ MRI-based neuroimaging

Antibodies

Antibodies used

Mouse anti-GFP Abcam Ab1218
Rabbit anti-GFP Abcam Ab290
Rabbit anti-DsRed Clontech 632496
Mouse anti-mCherry Clontech 632543
Goat anti-mouse Alexa 488 Life Technologies A11001
Goat anti-rabbit Alexa 488 Life Technologies A11008
Goat anti-rabbit Alexa 568 Life Technologies A11011
Goat anti-mouse Alexa 568 Life Technologies A11004

Validation

No custom-made antibodies were used in this study. All commercially available antibodies used have been previously validated for Nematostella (Tournière et al., 2020; Busengdal and Rentzsch, 2017; Richards and Rentzsch, 2015; Richards and Rentzsch, 2014; Nakanishi et al., 2012).The anti-DsRed, anti-mCherry and anti-GFP antibodies do not recognise endogenous proteins in Nematostella. These antibodies have been validated using stainings on wildtype animals which show no non-specific stainings.

Animals and other research organisms

Policy information about studies involving animals; ARRIVE guidelines recommended for reporting animal research, and Sex and Gender in Research

Laboratory animals

This study involved the use of wildtype Nematostella vectensis (laboratory strain CH2 x CH6), previously published transgenic and mutant lines [NvSoxB(2)::mOrange, NvPOU4::mCherry, NvMyHC1::homer-mCherry, NvFoxA::mOrange, NvElav1::mOrange, NvNcol3::mOrange2, NvPOU4-/-] as well as one new lines: NvPrdm14d::GFP. Male and Female adult animals of unknown age were used to produce eggs/sperm. Embryos were then utilized at various points up to 4 months depending on the experiment.

Wild animals

No wild animals were used in this study.

Reporting on sex

The sex of embryos and juveniles was not determined.

Field-collected samples

No field collected animals were used in this study.

Ethics oversight

Ethical approval is not required for work on Nematostella.

Note that full information on the approval of the study protocol must also be provided in the manuscript.

Flow Cytometry

Plots

Confirm that:

☒ The axis labels state the marker and fluorochrome used (e.g. CD4-FITC).

☒ The axis scales are clearly visible. Include numbers along axes only for bottom left plot of group (a 'group' is an analysis of identical markers).

☒ All plots are contour plots with outliers or pseudocolor plots.

☒ A numerical value for number of cells or percentage (with statistics) is provided.

Methodology

Sample preparation

Briefly, animals were dissociated in 0.25% Trypsin (Gibco, 27250018) in Ca- Mg- free Nematostella medium (CMFNM) (154 mM NaCl, 3.6 mM KCl, 2.4 mM Na2SO4, 0.7 mM NaHCO3) supplemented with 6.6 mM EDTA , pH 7.6-7.8. Cells were centrifuged at 800 g for 10 minutes, resuspended in ice cold 0.5% BSA in CMFNM (pH 7.6-7.8), filtered through a 40 µM filter and stained with Hoechst 33342 (Thermo Fisher Scientific, 62249) at a 60 µg/ml at RT for 30 minutes. Samples were then diluted 1:1 with ice cold 0.5% BSA/CMFNM and stained with 60 µl/ml 7-AAD (BD, 559925) for > 20 minutes on ice.

Instrument	A BD FACSAria II with a 100 μm nozzle was used for FACS.
Software	BD FACSDiva was used during cell sorting.
Cell population abundance	For all FACS experiements, the cells were resorted and >80% purity was obtained in all cases.
Gating strategy	For determining gates both single stain only and fluorescence minus one control were used.

☒ Tick this box to confirm that a figure exemplifying the gating strategy is provided in the Supplementary Information.
